# Widespread selenium deficiency in the brain of cases with Huntington's disease presents a new potential therapeutic target

**DOI:** 10.1016/j.ebiom.2023.104824

**Published:** 2023-10-10

**Authors:** Melissa Scholefield, Stefano Patassini, Jingshu Xu, Garth J.S. Cooper

**Affiliations:** a Centre for Advanced Discovery & Experimental Therapeutics, Division of Cardiovascular Sciences, School of Medical Sciences, Faculty of Biology, Medicine and Health, The University of Manchester, Manchester Academic Health Science Centre, Manchester, M19 9NT, United Kingdom; b School of Biological Sciences, Faculty of Science, University of Auckland, Private Bag 92 019, Auckland, 1142, New Zealand

**Keywords:** Neurodegeneration, Huntington's disease, Selenium homeostasis, Selenium deficiency, Mass spectrometry, Metallomics

## Abstract

**Background:**

Huntington or Huntington's disease (HD) is an autosomal dominant neurodegenerative disease characterised by both progressive motor and cognitive dysfunction; its pathogenic mechanisms remain poorly understood and no treatment can currently slow, stop, or reverse its progression. There is some evidence of metallomic dysfunction in limited regions of the HD brain; we hypothesised that these alterations are more widespread than the current literature suggests and may contribute to pathogenesis in HD.

**Methods:**

We measured the concentrations of eight essential metals (sodium, potassium, magnesium, calcium, iron, zinc, copper, and manganese) and the metalloid selenium across 11 brain regions in nine genetically confirmed, clinically manifest cases of HD and nine controls using inductively-coupled plasma mass spectrometry. Case–control differences were assessed by non-parametric Mann–Whitney U test (p < 0.05), risk ratios, E-values, and effect sizes.

**Findings:**

We observed striking decreases in selenium levels in 11 out of 11 investigated brain regions in HD, with risk ratios and effect sizes ranging 2.3–9.0 and 0.7–1.9, respectively. Increased sodium/potassium ratios were observed in every region (risk ratio = 2.5–8.0; effect size = 1.2–5.8) except the substantia nigra (risk ratio = 0.25; effect size = 0.1). Multiple regions showed increased calcium and/or zinc levels, and localised decreases in iron, copper, and manganese were present in the globus pallidus, cerebellum, and substantia nigra, respectively.

**Interpretation:**

The observed metallomic alterations in the HD brain may contribute to several pathogenic mechanisms, including mitochondrial dysfunction, oxidative stress, and blood–brain barrier dysfunction. Selenium supplementation may represent a potential, much-needed therapeutic pathway for the treatment of HD that would not require localised delivery in the brain due to the widespread presence of selenium deficiency in regions that show both high and low levels of neurodegeneration.

**Funding:**

In Acknowledgments, includes the Lee Trust, the Endocore Research Trust, 10.13039/100006500Cure Huntington's Disease Initiative, the 10.13039/100012744Oakley Mental Health Research Foundation, the 10.13039/501100000265Medical Research Council (MRC), the New Zealand Neurological Foundation, and others.

**Research in context**

**Evidence before this study**

In order to review the previous literature on metallomic dysfunction in Huntington disease (HD), we searched Pubmed for articles published up until January 8, 2023 for the following keywords: “Huntington disease”, “Huntington's brain”, “metal dyshomeostasis”, “metallomics”, “essential metals”, “sodium”, “magnesium”, “potassium”, “calcium”, “manganese”, “iron”, “copper”, “zinc”, and “selenium”. A few reports showed limited alterations in HD brain metal levels, including localised decreases in selenium, copper, and manganese, increases in sodium and zinc, and both increases and decreases in iron; however, no previous studies investigating all of these metals simultaneously across several regions of the HD brain in the same cohort could be found. This study aimed to investigate whether previously reported changes could be seen in end-stage post-mortem HD brains and to determine whether these changes were localised to regions with high levels of neurodegeneration or whether they could be found throughout the HD brain. Such alterations may provide evidence of metal-related pathogenic mechanisms in HD or suggest potential therapeutic targets.

**Added value of this study**

This study investigated the concentrations of eight essential metals and selenium across 11 regions of the HD brain—including regions with high, moderate, and relatively low levels of neurodegeneration—using a highly precise and sensitive methodology: inductively coupled plasma mass spectrometry (ICP-MS). Using these methods, we were able to identify widespread increases in sodium/potassium ratios, affecting every investigated region except the substantia nigra. We were also able to identify more localised alterations in calcium, manganese, iron, copper, and zinc. Most strikingly, we observed decreased selenium levels in every single investigated region; these data provide evidence to support the presence of widespread metallic dyshomeostasis in the HD brain, which may play a role in the pathogenesis of the disease.

**Implications of all the available evidence**

The findings described in this study provide evidence for widespread metallic dyshomeostasis in the HD brain, affecting not only regions with high levels of neurodegeneration, but also areas with more limited neuronal loss. In particular, selenium deficiency appears to be extensive throughout the HD brain—with further studies determining the time course, cause, and downstream effects of selenium alterations in HD, such selenium deficiency may present a potential therapeutic target for the treatment of this disease.

## Introduction

Huntington disease (Huntington's disease; HD) is a neurodegenerative disorder caused by an autosomal dominant mutation in the *HTT* gene—an expansion of the trinucleotide repeat located on exon-1 of *HTT* to 36 or more polyglutamine (CAG) repeats—leading to the production of mutated Huntingtin (mHTT). This mutation has been estimated to be present in as many as 139 per 100,000 person-years,^1^ with clinically manifest HD having a prevalence of around 10.6–13.7 per 100,000 person-years,^2^ based on studies of Western populations. A lower incidence of 2.4 and 0.25 per 100,000 for clinically manifest HD individuals has been reported in Asian and African populations, respectively.^3^ Despite the devastating impact of this disease, there are currently no disease-slowing or curative treatments for HD, making the identification of its pathogenic mechanisms and potential therapeutic targets a matter of the utmost importance. The mechanisms by which the CAG expansion mutation in *HTT* results in the pathogenesis of the disease remain poorly understood, and clinical trials aiming to lower levels of HTT and/or mHTT have thus far reported little to no clinical benefit. As such, investigations identifying the mechanisms by which neurodegeneration and symptoms occur in HD need to look beyond studies of only mHTT itself.

Metallomic dysfunction has been reported in the HD brain, including decreased copper (Cu) in the anterior cingulate gyrus (CG),^4^ decreased manganese (Mn) in the superior frontal gyrus and anterior CG,^4^ widespread increases in sodium (Na),^5^ increased Zn in the pallidum and putamen (PUT),^4^ multi-regional decreases in Se,^6^ and both increases and decreases in iron (Fe) across several regions.^4^^,^^7^, ^8^, ^9^, ^10^ Such perturbations may contribute to pathogenesis in HD, as metals such as Mn, Zn, Se, and Cu are essential co-factors for antioxidative molecules, and other elements such as Na are essential to maintaining gradients important for the normal physiological functioning of the electron transport chain (ETC) in order to facilitate energy production by the mitochondria. To date, many studies of metallomic dysfunction in HD have been limited to studying only a few specific metals or brain regions or have used methodologies that preclude the precise quantification of the metals being studied (e.g., Magnetic Resonance Imaging; MRI). The current study aimed to address these potential limitations by performing a multi-regional study of 11 HD brain regions using inductively-coupled plasma mass spectrometry, which allowed for the very sensitive, accurate, and precise quantification of nine essential metals in regions traditionally considered to be severely affected, moderately affected, and relatively spared in HD, so as to determine whether metallomic changes are widespread or constrained to areas of the brain showing prominent neurodegeneration.

## Methods

### Reagents

Except where otherwise stated, all reagents were obtained from Sigma–Aldrich (UK).

### Acquisition of human brain tissues from HD cases and controls

Brain tissues from nine HD cases and nine controls were obtained from the NZ Neurological Foundation Douglas Human Brain Bank at the University of Auckland, donated by individuals living in New Zealand. Tissues from 11 different regions were identified and dissected by a consultant neuropathologist; these were the cerebellum (CB), substantia nigra (SN), motor cortex (MCX; Brodmann area 4), middle frontal gyrus (MFG; Brodmann area 46), middle temporal gyrus (MTG; Brodmann area 21), sensory cortex (SCX; Brodmann area 1), cingulate gyrus (CG; Brodmann area 24), hippocampus (HP; Brodmann area 35), entorhinal cortex (ENT; Brodmann area 28), globus pallidus (GP), and putamen (PUT).

### Diagnosis and severity of human cases

All cases had a genetically confirmed diagnosis of HD. Cases were graded according to the Vonsattel criteria^11^ whereas controls were confirmed to be free of HD or a clinical or neuropathological diagnosis of any other neurodegenerative disease (e.g., Alzheimer disease, Parkinson disease, etc.). Medical reports provided further characterisation of cases and controls, including data detailing causes of death, CAP scores, Vonsattel grades, number of CAG repeats, and brain weight (see Supplementary Table S1). All HD cases were clinically manifest at the time of death. CAP scores were determined according to the standardised equation proposed by Warner et al.^12^

### Tissue dissection

Brain tissue was stored at −80 °C until analysis. On the experimental day, tissues were thawed slightly on ice to allow dissection. Tissues were sectioned into 50 mg (±5%) wet weight for ICP–MS using a metal-free ceramic scalpel and placed into ‘Safe-Lok’ microfuge tubes (Eppendorf AG; Hamburg, Germany). The use of a ceramic scalpel prevented contamination from trace metals during sectioning.

### ICP-MS

Freshly dissected samples were briefly centrifuged before being dried for 6 h to a constant weight (approximately 10 mg; individual sample wet and dry weights are available in Supplementary Material B) using a Savant Speedvac™ (Thermo Fisher Scientific, Massachusetts, USA). Nitric acid digestion of samples was then performed in a heat block, along with digestion blanks containing nitric acid alone (see Supplementary Material B). Following digestion, samples were refrigerated overnight at 4 °C before undergoing ICP-MS analysis with a 7700x ICP-MS spectrometer (Agilent, Santa Clara, USA) equipped with a MicroMist nebulizer and Scott double-pass spray chamber (Glass Expansion, Melbourne, Australia), and nickel sample and skimmer cones. Samples were separated into batches of either one or two regions, with multi-element calibration using calibration standard dilutions and periodic quality controls included for each batch (see Supplementary Material B for all raw data and values for blanks and standard curves). All elements were standardised against scandium, with the exception of zinc and selenium which were standardised against germanium. All regions were run in triplicate.

The Manufacturer's recommendations were followed for selection of operation mode, integration times, and internal standard assignments. Samples were introduced to the instrument using an integrated autosampler (Agilent, Santa Clara, USA). The concentrations of eight essential metals (Na, Mg, K, Ca, Mn, Fe, Cu, and Zn) and the metalloid Se were determined. All elements were analysed using helium as the collision gas; selenium was analysed in high-energy helium mode (10 ml/min helium) due to its potential state as a polyatomic ion, and all other elements were analysed using standard helium mode (5.0 ml/min helium). Results were excluded from analysis where the highest blank value for any given analyte during a run was ≥15% of that of the lowest sample value.

### ICP-MS data analysis and statistics

Case–control age, PMD, and brain weight were compared using the Mann–Whitney U test. Mean metal values and Na/K ratios (±standard deviation (SD) and with 95% confidence intervals (CI)); taken from the mean of the three triplicate runs) were calculated and differences between cases and controls determined by non-parametric Mann–Whitney U test due to the small sample size. Shannon diversity indices (S-values AKA surprisal scores) were also calculated by taking the negative base 2 log of the p value. Confidence intervals were calculated using the following equation:

(1) CI=SE∗Z(0.95)

where SE = the standard error and Z (0.95) = the z-score corresponding to a confidence level of 0.95. Mann–Whitney U calculations were performed using GraphPad v8.1.2 (Prism; La Jolla, CA). p values < 0.05 were considered significant. Comparisons of metal levels across different regions in only control or only HD brains were also carried out using Kruskal–Wallis tests in GraphPad v.8.1.2.

Individual profiles were generated for each case and control in order to determine their contribution to any observed case–control differences. This was carried out by determining the mean deviation of each sample from the control mean for each metal across all 11 regions and for all metals across each region, using the following equation to calculate the deviation from the control mean for each metal in each region for each individual sample:

(2) Deviationfromcontrolmean=C/M

where C = concentration of metal in sample and M = mean control value. Z-scores were then calculated using the equation:

(3) z−score=(x−μ)/σ

where ***χ*** = the deviation from the control mean of the sample, ***μ*** = the population mean, and ***σ*** = the population standard deviation. Samples with z-scores >2 were eliminated from analyses to if this altered the results of the case–control statistical tests. By doing this, we were able to determine whether any particular samples over-contributed to case–control differences and could analyse the dataset without these samples to determine whether case–control differences changed, as determined by Mann–Whitney U tests. The individual profiles generated can be found in Supplementary Material Figure S3.

### Sensitivity analyses

In order to assess whether the interpretation of the data obtained in the current study was appropriate and robust, a sensitivity analysis was performed for every significant (p < 0.05) case–control difference in metal levels. For both individual runs and the mean values of all three replicate runs taken together, the risk ratio (RR), E-value, and effect size were determined. An explanation of these is given below.

The risk ratio is used to compare the risk of a ‘health event’ between different groups (in our case, to compare HD cases and controls); it is determined by the following equation:

(4) $$RR=\left(\frac{a}{b}\right)/\left(\frac{c}{d}\right)$$

where ***a*** = the number of case values > 95% upper CI limit of the controls (or <95% lower CI limit where significant *decreases* were observed in cases), ***b*** = number of cases, ***c*** = number of control values > 95% upper CI limit of the controls (or <95% lower CI limit where significant *decreases* were observed in cases), and ***d*** = number of controls. Risk ratios of >3 were considered to be robust. In the case of null values in the calculation of risk ratios, the null values were assigned a value of 0.5.

E-values were calculated for risk ratios as well as for the upper and low confidence limits of the risk ratios. The E-value defines the minimum strength of association, on the risk ratio scale, that a potential confounder would have to have with both a treatment (e.g., metal/Se levels) and an outcome (e.g., an increased risk of HD) to explain away an observed treatment–outcome association (i.e., the observed association between concentrations of a metal/Se and an increased risk of HD), while taking into account measured covariates (here including age, sex, PMD, and brain weight). The higher the E-value, the stronger the confounding required to nullify the treatment–outcome association. The E-value was calculated using Equation^5^:

(5) Evalue=RR+sqrt(RRx(RR−1))

in the calculation of E-values for RR < 1, the inverse of the risk ratio was first taken. E-values were also calculated for the confidence intervals of the risk ratios; if the RR < 1, then the lower limit of the CI (LL) was used to calculate this, whereas if the RR < 1, the upper limit of the CI (UL) was used. For the former, if the LL ≤ 1, then the E-value was determined to be 1; if the LL > 1, then the E-value was calculated according to Equation 5, with RR substituted by LL. For the latter, if the UL ≥ 1, then the E-value was determined to be 1; if the UL < 1, then the inverse of the UL was taken, and Equation 5 used to calculate the E-value, with RR substituted by the inverse UL.

The effect size describes the *strength* of the relationship observed between variables, rather than indicating whether differences are present due to chance or otherwise. Determination of effect sizes can indicate where significantly altered (p < 0.05) variables have a negligible influence on an outcome, or conversely where variables found to be non-significant (p < 0.05) in traditional statistical testing have a large contribution towards an outcome; the latter may occur where statistical power is low due to small sample sizes. The effect size was here determined using Glass’ Delta:

(6) $$Glass^{\prime }\Delta =\left(M_{1}-M_{2}\right)/\sigma control$$

where M_1_ = mean case value, M_2_ = mean control value, and σcontrol = standard deviation of the control group; M_1_ and M_2_ were reversed in case of significant decreases. Glass' delta was used as the group sample sizes were equal, but their standard deviations were unequal. Effect size values 0.2–0.5 were considered small, values between 0.50 and 0.80 were considered of medium size, values between 0.80 and 1.30 were considered large, and effect sizes >1.30 were considered very large.

When analysing potential cause–effect relationships between any significant case–control alterations in analytes observed in the current study and the development of HD, the Bradford–Hill considerations for causation were considered; an example of the application of the application of these considerations is given for the obtained Se data in Supplementary Material A.

### Ethics

All donors (and/or their families, when applicable) gave informed consent for the donation of the brain tissues used in this study. The collection of tissues was approved by the Auckland Human Participants Ethics Committee and the study was reviewed, approved, and carried out in compliance with the approval made by Manchester REC (09/H0906/52 + 5).

### Role of funders

The funders had no role in the study design, data collection, data analyses, interpretation, or writing of this report.

### Sex and gender equity in research

This study included both male and female donor samples, with sex-matching between cases and controls. Data on sex were collected and provided by the NZ Neurological Foundation Douglas Human Brain Bank upon provision of samples.

## Results

### Patient characteristics

Tissues were obtained from 11 regions from nine genetically confirmed HD cases and nine HD-free controls. Cases and controls showed no significant differences with respect to sex (three female and six male), age (mean 67.4 vs. 65.2 years, respectively; p = 0.6) or PMD (mean 10.6 vs. 11.2 h, respectively; p = 0.7; Table 1). Brain weight was significantly lower in HD cases (mean 1104 g) compared to controls (mean 1322 g; p = 0.018); this is likely due to extensive neurodegeneration in the HD brains. The neuropathological classification of HD cases ranged across Vonsattel grades 1–4, with an average grade of two. CAP scores in HD cases ranged from 123.3 to 154.5. The most common cause of death in HD cases was bronchopneumonia, whereas the most common cause in controls was ischaemic heart disease. All data that could be obtained for the supplied samples is available in Supplementary Table S1, including individual information on age, sex, PMD, brain weight, HD Vonsattel grade (for cases), CAP scores, CAG repeat length, and cause of death.

**Table 1:** Cohort characteristics.

	Controls (n = 9)	HD cases (n = 9)	p value
Male Sex, n	6	6	1.0
Age	67 ± 9.7 (49–81)	65 ± 10.3 (51–80)	0.6
PMD (h)	10.6 ± 3.2 (6.5–15)	11.2 ± 2.9 (7–15)	0.7
Whole Brain Weight (g)	1322 ± 95.8 (1210–1495)	1104 ± 216.1 (787–1497)	**0**.**018**

Comparison of HD case and control variables. All variables are means ± SD (range); p values for significance of between-group differences were calculated by Mann–Whitney U test. PMD = post-mortem delay. LC = locus coeruleus; MED = medulla oblongata; PVC = primary visual cortex (occipital lobe); MCX = motor cortex.

### ICP-MS metals analysis

**Fig. 1:**
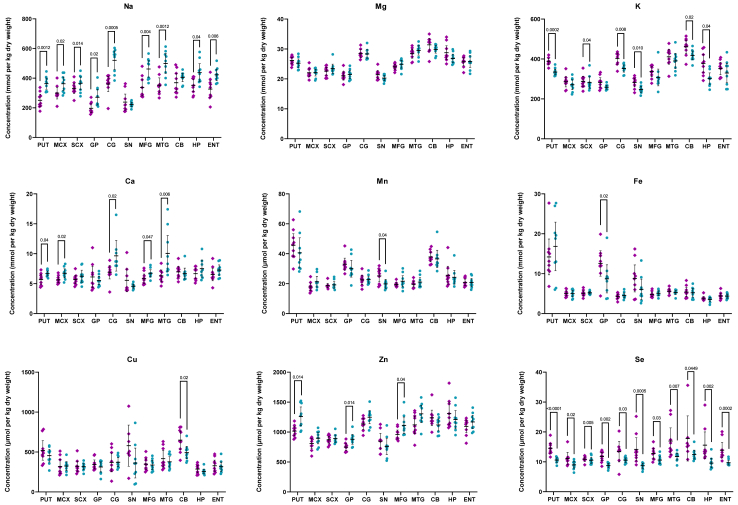
Concentrations of Eight Essential Metals and Selenium in HD Cases vs Controls. Data shown are mean ± 95% confidence intervals. Each data point denotes the mean of three biological replicates. Case–control differences determined by Mann–Whitney U test; p < 0.05 was considered significant; n = 7–9. Purple diamonds represent controls and blue circles represent HD cases; error bars denote ±95% CI. C = Controls; CB = cerebellum; CG = Cingulate gyrus; ENT = Entorhinal cortex; GP = Globus pallidus; HD = HD cases; HP = Hippocampus; MCX = Motor cortex; MFG = Middle frontal gyrus; MTG = Middle temporal gyrus; PUT = Putamen; SCX = Sensory cortex; SN = Substantia nigra.

Levels of eight essential metals and Se were measured in dry tissue from 11 brain regions, ranging from those severely affected by neurodegeneration in HD to those considered to be relatively spared by the disease, in nine HD cases and nine controls using ICP-MS. Concentrations of individual metals for each region, case–control p values (determined by nonparametric Mann–Whitney U test due to small sample size; t-test results also provided in Supplementary Table S2 for completeness) and S-values, risk ratios, E-values, and effect sizes are shown in Figure 1 and Table 2, Table 3, Table 4, Table 5, Table 6, Table 7, Table 8, Table 9, Table 10, Table 11, Table 12. For completeness, an inter-regional analysis of analyte concentrations in cases and in controls is included in Supplementary Material A.

**Table 2:** ICP–MS analysis of HD and control PUT.

	Controls	Cases	p value	S-value	E-value	Risk Ratio (RR)	RR CI E-values	Effect size
Na	250.7 ± 53.3 (209.7–291.6)	362.9 ± 48.8 (325.4–400.4)	0.0012	9.7	8.5	4.5 (1.3–15.3)	1.9	2.1
Mg	26.1 ± 1.5 (25.0–27.3)	25.3 ± 1.9 (23.8–26.7)	0.4	1.3	3.4	2.0 (0.5–8.3)	1.0	0.6
K	388.7 ± 20.0 (373.3–404.0)	334.6 ± 18.9 (320.1–349.2)	0.0002	12.3	17.5	9.0 (1.4–57.1)	2.1	2.7
Ca	5.7 ± 1.0 (4.9–6.5)	6.7 ± 0.5 (6.3–7.1)	0.04	4.6	3.4	2.0 (0.5–8.3)	1.0	1.0
Mn	45.7 ± 10.0 (38.0–53.4)	40.6 ± 13.1 (30.5–50.6)	0.3	1.7	3.4	5.0 (0.7–34.7)	1.0	0.5
Fe	14.2 ± 5.8 (9.7–18.7)	16.8 ± 7.9 (10.7–22.9)	0.5	1.0	9.5	5.0 (0.7–34.7)	1.0	0.4
Cu	520.3 ± 159.8 (397.5–643.1)	455.0 ± 102.9 (376.0–534.1)	0.7	0.5	9.5	1.0 (0.2–5.6)	1.0	0.4
Zn	1016.1 ± 100.7 (938.7–1093.5)	1261.3 ± 205.4 (1103.5–1419.1)	0.014	6.2	13.5	7.0 (1.0–12.5)	1.0	2.4
Se	14.6 ± 2.2 (12.9–16.2)	10.6 ± 1.0 (9.8–11.3)	<0.0001	>13.3	17.5	9.0 (1.4–57.1)	2.1	1.9

**Table 3:** ICP–MS analysis of HD and control MCX.

	Controls	Cases	p–value	S-value	E-value	Risk Ratio (RR)	RR CI E-values	Effect size
Na	299.4 ± 51.7 (259.6–339.1)	362.3 ± 58.8 (317.1–407.5)	0.02	5.6	4.4	2.5 (0.6–9.7)	1.0	2.1
Mg	22.0 ± 1.7 (20.7–33.3)	22.1 ± 1.5 (21.0–23.2)	0.6	0.7	1.0	1.0 (0.2–5.6)	1.0	0.05
K	285.5 ± 33.2 (260.0–311.0)	271.3 ± 32.2 (246.5–296.1)	0.4	1.3	2.4	1.5 (0.3–6.9)	1.0	0.4
Ca	5.6 ± 0.7 (5.1–6.1)	6.7 ± 1.0 (6.0–7.5)	0.02	5.6	6.5	3.5 (1.0–12.5)	1.0	1.6
Mn	17.8 ± 3.3 (15.2–20.3)	21.3 ± 4.6 (17.7–24.9)	0.08	3.6	5.4	3.0 (0.8–12.5)	1.0	1.1
Fe	5.1 ± 0.8 (4.4–5.7)	5.0 ± 1.0 (4.3–5.8)	0.9	0.2	1.0	1.0 (0.2–5.6)	1.0	0.04
Cu	319.8 ± 112.2 (233.6–406.0)	324.6 ± 71.2 (269.9–379.3)	0.5	1.0	3.4	0.5 (0.05–4.6)	1.0	0.04
Zn	808.4 ± 109.1 (724.6–892.2)	897.3 ± 122.9 (802.8–991.8)	0.2	2.3	2.7	1.7 (0.6–5.6)	1.0	0.8
Se	11.1 ± 2.5 (9.2–13.0)	9.0 ± 1.4 (7.9–10.0)	0.02	5.6	4.4	2.5 (3.0–9.7)	5.4	0.9

**Table 4:** ICP–MS analysis of HD and control SCX.

	Controls	Cases	p value	S-value	E-value	Risk Ratio (RR)	RR CI E-values	Effect size
Na	335.7 ± 52.2 (295.6–375.8)	404.1 ± 48.2 (367.0–441.2)	0.014	6.2	4.4	2.5 (0.6–9.7)	1.0	1.3
Mg	24.1 ± 1.8 (22.8–25.5)	24.1 ± 2.1 (22.5–25.7)	0.8	0.3	2.4	1.5 (0.3–6.9)	1.0	0.009
K	322.6 ± 28.7 (294.0–351.3)	282.0 ± 50.3 (243.3–320.7)	0.04	4.6	6.5	3.5 (1.0–12.5)	1.0	1.1
Ca	5.7 ± 0.7 (5.0–6.4)	6.2 ± 1.3 (5.2–7.2)	0.4	1.3	7.5	4.0 (0.5–29.2)	1.0	0.6
Mn	20.0 ± 1.8 (18.2–21.7)	19.9 ± 3.7 (17.1–22.8)	0.7	0.5	3.4	2.0 (0.5–29.5)	1.0	0.02
Fe	5.4 ± 0.6 (4.8–6.0)	5.1 ± 0.6 (4.6–5.5)	0.4	1.3	1.0	1.0 (0.3–3.7)	1.0	0.4
Cu	360.4 ± 94.0 (266.4–454.3)	295.9 ± 39.2 (265.8–326.1)	0.3	1.7	5.4	3.0 (0.4–23.7)	1.0	0.5
Zn	913.8 ± 74.4 (839.4–988.2)	967.4 ± 140.5 (859.4–1075.3)	0.4	1.3	1.0	0.75 (0.3–3.7)	1.0	0.6
Se	12.5 ± 1.3 (11.2–13.8)	10.1 ± 1.5 (9.0–11.2)	0.005	7.6	7.5	4.0 (0.9–10.9)	1.0	1.4

**Table 5:** ICP–MS analysis of HD and control GP.

	Controls	Cases	p value	S-value	E-value	Risk Ratio (RR)	RR CI E-values	Effect size
Na	197.0 ± 38.9 (167.1–226.9)	271.4 ± 68.8 (218.6–324.3)	0.02	5.6	4.4	2.5 (0.6–9.7)	1.0	1.9
Mg	21.0 ± 1.8 (19.6–22.3)	21.5 ± 1.7 (20.2–22.8)	0.7	0.5	7.5	4.0 (0.4–23.7)	1.0	0.3
K	282.6 ± 30.6 (259.1–306.2)	258.3 ± 12.3 (248.4–268.3)	1.3	0.4	2.0	0.8 (0.4–4.3)	1.0	0.8
Ca	6.1 ± 2.3 (4.4–7.9)	5.5 ± 0.9 (4.8–6.2)	1.0	0	7.5	0.25 (0.01–3.7)	1.0	0.3
Mn	33.0 ± 5.2 (29.0–37.0)	30.0 ± 7.3 (24.4–35.6)	0.1	3.3	1.0	1.0 (0.5–29.2)	1.0	0.6
Fe	12.6 ± 4.2 (9.4–15.8)	8.7 ± 4.6 (5.2–12.2)	0.02	5.6	13.5	7.0 (1.1–45.9)	1.4	0.9
Cu	344.3 ± 73.3 (287.9–400.6)	312.8 ± 82.1 (249.7–375.9)	0.8	0.3	2.4	1.5 (0.3–6.9)	1.0	0.4
Zn	749.0 ± 73.1 (692.8–805.2)	878.4 ± 106.3 (796.7–960.1)	0.014	6.2	6.5	3.5 (1.0–12.5)	1.0	1.8
Se	11.8 ± 1.9 (10.3–13.2)	8.8 ± 0.9 (8.1–9.5)	0.002	9.0	8.5	4.5 (1.3–15.3)	1.9	1.5

**Table 6:** ICP–MS analysis of HD and control CG.

	Controls	Cases	p value	S-value	E-value	Risk Ratio (RR)	RR CI E-values	Effect size
Na	360.7 ± 67.4 (308.9–412.5)	520.7 ± 81.0 (458.4–583.0)	0.0005	11.0	7.5	4.0 (1.2–13.9)	1.7	2.4
Mg	27.4 ± 1.5 (24.7–30.1)	28.3 ± 1.8 (26.9–29.7)	0.9	0.2	3.9	0.4 (0.05–4.0)	1.0	0.6
K	401.8 ± 34.0 (375.7–428.0)	352.1 ± 23.1 (334.4–369.9)	0.008	7.0	5.7	3.1 (0.9–10.9)	1.0	1.5
Ca	6.9 ± 0.8 (5.8–8.0)	9.6 ± 3.0 (7.3–12.0)	0.02	5.6	13.0	6.8 (1.02–44.7)	1.2	1.9
Mn	22.5 ± 3.3 (19.5–25.5)	22.6 ± 3.1 (20.3–25.0)	0.8	0.2	3.4	2.0 (0.2–18.3)	1.0	0.04
Fe	4.3 ± 0.6 (3.7–4.9)	4.5 ± 0.8 (3.9–5.2)	0.6	0.7	1.0	1.0 (0.2–5.6)	1.0	0.4
Cu	370.0 ± 133.8 (252.4–487.6)	372.1 ± 75.0 (314.4–429.7)	0.8	0.3	6.2	0.3 (0.04–2.3)	1.0	0.02
Zn	1052.3 ± 110.8 (843.0–1261.7)	1247.5 ± 144.0 (1136.9–1358.2)	0.1	3.3	9.5	5.0 (0.7–34.7)	1.0	1.8
Se	13.4 ± 2.9 (10.2–13.6)	10.6 ± 1.0 (9.8–11.3)	0.03	5.1	4.8	2.7 (0.3–20.8)	1.0	0.7

**Table 7:** ICP–MS analysis of HD and control SN.

	Controls	Cases	p value	S-value	E-value	Risk Ratio (RR)	RR CI E-values	Effect size
Na	238.5 ± 70.6 (184.2–292.8)	222.0 ± 18.8 (207.6–236.4)	0.9	0.2	7.5	0.25 (0.01–3.7)	1.0	0.2
Mg	21.4 ± 1.7 (20.1–22.7)	20.3 ± 1.1 (19.4–21.1)	0.1	3.3	2.7	1.7 (0.6–5.0)	1.0	0.7
K	282.4 ± 28.1 (260.8–304.0)	247.7 ± 19.2 (233.0–262.5)	0.010	6.6	6.5	3.5 (1.0–12.5)	1.0	1.2
Ca	5.5 ± 2.3 (3.8–7.3)	4.6 ± 0.4 (4.3–4.9)	0.9	0.2	3.9	0.4 (0.05–4.0)	1.0	0.4
Mn	25.2 ± 5.2 (21.2–29.2)	19.8 ± 3.9 (16.8–22.8)	0.04	4.6	3.4	2.0 (0.9–6.3)	1.0	1.0
Fe	8.8 ± 4.4 (5.4–12.2)	6.1 ± 3.3 (3.5–8.6)	0.2	2.3	4.4	2.5 (0.6–9.7)	1.0	0.6
Cu	579.5 ± 278.6 (365.3–793.6)	361.9 ± 243.8 (174.4–549.3)	0.09	3.5	8.8	4.7 (0.7–30.4)	1.0	0.8
Zn	846.7 ± 172.4 (714.2–979.3)	761.0 ± 176.9 (625.1–897.0)	0.4	1.3	2.4	1.5 (0.3–6.9)	1.0	0.5
Se	14.2 ± 5.1 (10.3–18.1)	8.7 ± 1.1 (7.9–9.5)	0.0005	11.0	8.5	2.7 (1.3–15.3)	1.9	1.1

**Table 8:** ICP–MS analysis of HD and control MFG.

	Controls	Cases	p value	S-value	E-value	Risk Ratio (RR)	RR CI E-values	Effect size
Na	337.6 ± 74.4 (280.4–394.8)	462.0 ± 72.6 (406.3–517.8)	0.004	8.0	6.5	3.5 (1.0–12.5)	1.0	1.7
Mg	24.0 ± 1.0 (23.2–24.8)	24.9 ± 1.7 (23.6–26.2)	0.2	2.3	4.4	2.5 (0.6–9.7)	1.0	0.9
K	337.0 ± 32.7 (311.9–362.1)	307.5 ± 54.9 (265.3–349.7)	0.1	3.3	6.5	3.5 (1.0–12.5)	1.0	0.9
Ca	5.8 ± 1.0 (5.0–6.6)	6.7 ± 0.8 (6.2–7.3)	0.047	4.4	5.5	3.0 (0.8–11.1)	1.0	0.9
Mn	19.5 ± 1.5 (18.3–20.6)	21.6 ± 4.8 (17.9–25.3)	0.3	1.7	7.6	4.0 (0.5–29.2)	1.0	1.4
Fe	4.8 ± 0.7 (4.2–5.3)	5.1 ± 0.8 (4.5–5.7)	0.4	1.3	3.4	2.0 (0.5–8.3)	1.0	0.4
Cu	347.9 ± 83.6 (283.6–412.1)	337.0 ± 72.5 (281.3–392.8)	0.9	0.2	2.4	1.5 (0.3–6.9)	1.0	0.1
Zn	958.9 ± 97.8 (883.7–1034.1)	1111.8 ± 180.1 (973.4–1250.2)	0.04	4.6	4.4	2.5 (0.6–9.7)	1.0	1.6
Se	12.8 ± 2.2 (11.1–14.4)	10.6 ± 1.5 (9.4–11.7)	0.03	5.1	3.4	2.0 (0.6–4.9)	1.0	1.0

**Table 9:** ICP–MS analysis of HD and control MTG.

	Controls	Cases	p value	S-value	E-value	Risk Ratio (RR)	RR CI E-values	Effect size
Na	352.6 ± 64.3 (303.1–402.0)	498.9 ± 86.9 (432.1–565.7)	0.0012	9.7	6.5	3.5 (1.0–12.5)	1.0	2.3
Mg	28.5 ± 2.0 (26.9–30.0)	29.5 ± 2.1 (27.9–31.1)	0.3	1.7	4.4	2.5 (0.6–9.7)	1.0	0.5
K	410.9 ± 47.8 (374.2–447.7)	390.3 ± 49.1 (352.6–428.1)	0.4	1.3	5.4	3.0 (0.4–23.7)	1.0	0.4
Ca	6.3 ± 1.2 (5.4–7.2)	8.1 ± 1.2 (7.2–9.1)	0.006	7.4	11.9	6.2 (1.0–40.2)	1.0	1.6
Mn	19.6 ± 2.4 (17.8–21.5)	21.0 ± 3.9 (18.0–24.0)	0.4	1.3	2.4	1.5 (0.3–6.9)	1.0	0.6
Fe	5.6 ± 0.7 (5.0–6.2)	5.3 ± 0.6 (4.8–5.8)	0.2	2.3	1.0	1.0 (0.2–5.6)	1.0	0.4
Cu	416.2 ± 135.6 (312.0–520.4)	377.2 ± 85.4 (311.6–442.8)	0.5	1.0	1.0	1.0 (0.2–5.6)	1.0	0.3
Zn	1119.7 ± 190.2 (973.5–1265.9)	1304.0 ± 185.4 (1161.5–1446.6)	0.09	3.5	2.7	1.7 (0.6–5.0)	1.0	1.0
Se	17.0 ± 5.7 (12.5–21.4)	11.8 ± 1.5 (10.6–12.9)	0.007	7.2	5.5	3.0 (0.8–11.1)	1.0	0.9

**Table 10:** ICP–MS analysis of HD and control CB.

	Controls	Cases	p value	S-value	E-value	Risk Ratio (RR)	RR CI E-values	Effect size
Na	369.6 ± 80.5 (307.7–431.4)	405.3 ± 37.8 (376.2–434.3)	0.3	1.7	5.4	0.3 (0.04–2.6)	1.0	0.4
Mg	31.4 ± 2.7 (29.3–33.5)	29.9 ± 1.6 (28.6–31.1)	0.1	3.3	1.0	1.0 (0.2–5.6)	1.0	0.6
K	460.9 ± 44.0 (427.1–494.7)	417.9 ± 28.7 (395.9–440.0)	0.02	5.6	11.5	6.0 (0.9–40.3)	1.0	1.0
Ca	7.0 ± 1.2 (6.0–7.9)	6.7 ± 1.2 (5.8–7.6)	0.4	1.3	2.4	1.5 (0.3–6.9)	1.0	0.2
Mn	37.9 ± 4.3 (34.6–41.2)	36.5 ± 7.5 (30.8–42.3)	0.4	1.3	4.3	2.5 (0.6–9.7)	1.0	0.3
Fe	5.4 ± 1.5 (4.2–6.6)	5.2 ± 1.4 (4.1–6.3)	0.8	0.3	1.0	1.0 (0.3–3.7)	1.0	0.1
Cu	648.3 ± 148.9 (533.8–762.7)	492.3 ± 100.8 (414.9–569.8)	0.02	5.6	7.5	4.0 (0.6–7.9)	1.0	1.0
Zn	1236.9 ± 168.6 (1107.3–1366.5)	1118.6 ± 131.4 (1017.6–1219.6)	0.2	2.3	5.4	3.0 (0.4–23.7)	1.0	0.7
Se	28.2 ± 8.1 (11.2–45.3)	18.0 ± 1.7 (11.8–24.3)	0.0449	4.5	4.1	2.3 (0.3–17.9)	1.0	0.7

**Table 11:** ICP–MS analysis of HD and control HP.

	Controls	Cases	p value	S-value	E-value	Risk Ratio (RR)	RR CI E-values	Effect size
Na	351.9 ± 55.0 (309.6–394.1)	437.6 ± 78.3 (377.4–497.7)	0.04	4.6	1.9	1.25 (0.5–3.2)	1.0	1.6
Mg	28.8 ± 3.0 (26.5–31.1)	26.9 ± 1.9 (25.4–28.3)	0.2	2.3	7.5	4.0 (0.5–29.2)	1.0	0.6
K	378.1 ± 58.7 (333.0–423.2)	301.7 ± 37.3 (272.1–330.4)	0.006	7.4	4.1	2.3 (0.9–6.3)	1.0	1.3
Ca	6.7 ± 1.1 (5.8–7.5)	10.3 ± 8.5 (3.8–16.9)	0.4	1.3	3.4	2.0 (0.5–8.3)	1.0	3.3
Mn	25.6 ± 7.8 (19.6–31.6)	24.3 ± 6.0 (19.6–28.9)	0.6	0.7	3.4	0.5 (0.05–4.6)	1.0	0.2
Fe	3.8 ± 0.6 (3.4–4.3)	3.6 ± 0.6 (3.1–4.1)	0.9	0.2	3.4	0.5 (0.05–4.6)	1.0	0.5
Cu	286.0 ± 59.5 (240.3–331.8)	256.1 ± 39.5 (225.7–286.5)	0.4	1.3	2.7	1.6 (0.6–5.0)	1.0	0.5
Zn	1310.1 ± 247.9 (1119.5–1500.6)	1208.6 ± 196.9 (1057.3–1359.9)	0.4	1.3	2.4	1.5 (0.3–6.9)	1.0	0.4
Se	15.6 ± 5.8 (11.2–20.0)	9.6 ± 2.1 (8.0–11.2)	0.002	9.0	15.5	8.0 (1.2–51.5)	1.7	1.0

**Table 12:** ICP–MS analysis of HD and control ENT.

	Controls	Cases	p value	S-value	E-value	Risk Ratio (RR)	RR CI E-values	Effect size
Na	326.3 ± 72.6 (270.5–382.1)	422.7 ± 50.0 (384.2–461.1)	0.006	7.4	4.0	2.3 (0.9–6.3)	1.0	1.3
Mg	25.7 ± 1.8 (24.4–27.1)	25.8 ± 2.5 (23.8–27.7)	0.7	0.5	1.0	1.0 (0.3–3.7)	1.0	0.03
K	351.8 ± 39.7 (321.3–382.4)	329.7 ± 63.9 (280.6–378.8)	0.6	0.7	2.0	1.3 (0.4–4.3)	1.0	0.6
Ca	6.5 ± 1.4 (5.5–7.6)	7.3 ± 1.1 (6.4–8.1)	0.3	1.7	2.4	1.5 (0.3–6.9)	1.0	0.5
Mn	20.9 ± 2.9 (18.7–23.1)	20.8 ± 3.5 (18.1–23.5)	0.9	0.2	2.4	0.7 (0.1–3.1)	1.0	0.03
Fe	4.4 ± 0.9 (3.7–5.1)	4.4 ± 0.7 (3.9–4.9)	0.8	0.3	1.0	1.0 (0.2–5.6)	1.0	0.03
Cu	327.8 ± 91.6 (257.8–398.2)	316.1 ± 75.1 (258.4–373.9)	0.9	0.2	1.0	1.0 (0.2–5.6)	1.0	0.1
Zn	1095.4 ± 137.8 (989.5–1201.3)	1169.2 ± 144.0 (1058.5–1279.9)	0.4	1.3	3.4	2.0 (0.2–18.3)	1.0	0.5
Se	13.9 ± 3.4 (11.3–16.5)	9.7 ± 1.0 (8.9–10.5)	0.0002	12.3	15.5	8.0 (1.2–51.5)	1.7	1.2

Na, Mg, K, Ca, and Fe concentrations given in mmol/kg dry weight; Mn, Cu, Zn, and Se concentrations given in μmol/kg dry weight. Data shown are means ± SD (95% CI); n = 7–9. Case–control differences determined by Mann–Whitney U test; values in bold denote significant case–control differences; p < 0**.**05 was considered significant. CB = cerebellum; CG = Cingulate gyrus; CI = Confidence interval; ENT = Entorhinal cortex; GP = Globus pallidus; HP = Hippocampus; MCX = Motor cortex; MFG = Middle frontal gyrus; MTG = Middle temporal gyrus; PUT = Putamen; RR = risk ratio; SCX = Sensory cortex; SN = Substantia nigra.

The most striking finding in this study was that of decreased Se in HD cases compared to controls in every investigated region. These decreases were strongest in the PUT (14.6 vs 10.6 μmol/kg dry weight; p < 0.0001; S-value >13.3; Table 2) and the ENT (13.9 vs 9.7 μmol/kg dry weight; p = 0.0002; S-value = 12.3; Table 12). As such, HD brains showed widespread Se decreases despite differing levels of neurodegeneration between regions. Despite the small size of the cohort employed here, effect sizes were large, ranging from 0.7 in the CG (Table 6) and CB (Table 10) to 1.9 in the PUT (Table 2), with RRs ranging from 2.3 to 9.0. Together, these data provide support for the presence of substantial Se deficiencies in the HD brain; however, the overlap of the RR confidence intervals over the 1.0 null value in several regions of the brain indicates the need for further validation in larger cohorts.

Also notable were widespread increases in Na in HD cases, which were observed in 9 out 11 investigated regions including the PUT (250.7 vs 362.9 mmol/kg dry weight; p = 0.0012; S-value = 9.7; Table 2), MCX (299.4 vs 362.3 mmol/kg dry weight; p = 0.02; S-value = 5.6; Table 3), SCX (335.7 vs 404.1 mmol/kg dry weight; p = 0.014; S-value = 6.2; Table 4), GP (197.0 vs 271.4 mmol/kg dry weight; p = 0.02; S-value = 5.6; Table 5), CG (360.7 vs 520.7 mmol/kg dry weight; p = 0.0005; S-value = 11.0; Table 6), MFG (337.6 vs 462.0 mmol/kg dry weight; p = 0.004; S-value = 8.0; Table 8), MTG (352.6 vs 498.9 mmol/kg dry weight; p = 0.0012; S-value = 9.7; Table 9), HP (351.9 vs 437.6 mmol/kg dry weight; p = 0.04; S-value = 4.6; Table 11), and ENT (326.3 vs 422.7 mmol/kg dry weight; p = 0.006; S-value = 7.4; Table 12). Risk ratios and effect sizes were large, ranging from 2.3 to 4.5 and 1.3 to 2.4, respectively, in regions showing p < 0.05; outside of these regions, effect sizes remained below 0.4. As with Se, these results indicate widespread alterations in Na that are not isolated to regions of the brain associated with high levels of HD-related neurodegeneration.

Although alterations in K were not as widespread as for Na, it showed substantial decreases in 6 out of the 11 investigated regions including the PUT (388.7 vs 334.6 mmol/kg dry weight; p = 0.0002; S-value = 12.3; effect size = 2.7; Table 2), SCX (332.6 vs 282.0 mmol/kg dry weight; p = 0.04; S-value = 4.6; effect size = 1.1; Table 4), CG (401.8 vs 352.1 mmol/kg dry weight; p = 0.008; S-value = 7.0; effect size = 1.5; Table 6), SN (282.4 vs 247.7 mmol/kg dry weight; p = 0.010; S-value = 6.6; effect size = 1.2; Table 7), CB (460.9 vs 417.9 mmol/kg dry weight; p = 0.02; S-value = 5.6; effect size = 1.0; Table 10), and HP (378.1 vs 301.7 mmol/kg dry weight; p = 0.006; S-value = 7.4; effect size = 1.3; Table 11). Effect sizes in other regions of the brain still showed small to large effect size, ranging from 0.4 in the MCX and MTG to 0.9 in the MFG; as such, further investigations of K in these regions with a larger cohort may remain of interest.

Multiple regions also showed alterations in Ca, with five showing significant increases in HD cases compared to controls, including the PUT (5.7 vs 6.7 mmol/kg dry weight; p = 0.04; S-value = 4.6; Table 2), MCX (5.6 vs 6.7 mmol/kg dry weight; p = 0.02; S-value = 5.6; Table 3), CG (6.9 vs 9.6 mmol/kg dry weight; p = 0.02; S-value = 5.6; Table 6), MFG (5.8 vs 6.7 mmol/kg dry weight; p = 0.047; S-value = 4.4; Table 8), and MTG (6.3 vs 8.1 mmol/kg dry weight; p = 0.006; S-value = 7.4; Table 9). Effect sizes in these regions were high, ranging from 0.9 in the MFG to 1.6 in the MTG and MCX. However, the effect size was highest in the HP, at 3.3, despite attaining a p-value <0.05; as such, Ca levels in this region may be of particular interest for further future studies. Effect sizes in other regions ranged from 0.2 in the CB to 0.6 in the SCX.

Three regions showed alterations in Zn, including significantly increased levels in the PUT (1016.1 vs 1261.3 μmol/kg dry weight; p = 0.014; S-value = 6.2; Table 2), GP (749.0 vs 878.4 μmol/kg dry weight; p = 0.014; S-value = 6.2; Table 5), and MFG (958.9 vs 1111.8 μmol/kg dry weight; p = 0.04; S-value = 4.6; Table 8) of HD cases. Effect sizes were large in all three of these regions, ranging from 1.6 in the MTG to 2.4 in the PUT. Effect sizes ranged from small to large in other regions of the brain, ranging from 0.4 in the HP to as high as 1.8 in the CG.

The SN was the only region in which significant changes in Mn were observed between cases and controls, with lower concentrations observed in HD cases (25.2 vs 19.8 μmol/kg dry weight; p = 0.04; S-value = 4.6; effect size = 1.0; Table 7). Similarly, only a single region showed significant alterations in Fe—in this case, the GP showed decreased concentrations in HD cases (12.6 vs 8.7 mmol/kg dry weight; p = 0.02; S-value = 5.6; effect size = 0.9; Table 5). Cu levels also only showed one significant difference, with decreased concentrations present in the HD CB compared to controls (648.3 vs 492.3 μmol/kg dry weight; p = 0.02; S-value = 5.6; effect size = 1.0; Table 10). Effect sizes ranged from very small (0.02; CG) to large (0.8; SN) for Cu, very small (0.04; CG) to high (1.4; MFG) for Mn, and very small (0.03; ENT) to moderate (0.6; SN) for Fe in other brain regions. There were no significant changes in Mg levels between cases and controls in any investigated brain region; effect sizes ranged from very small (0.009; SCX) to large (0.9; MFG).

Taken together, these results provide support for several substantial metallomic alterations in the HD brain, with many case–control differences showing high risk ratios and effect sizes despite the small sample size. However, the results also indicate a need for further studies in larger cohorts to strengthen the robustness of observed findings and to investigate larger effect sizes where they occur alongside high p values.

### Na/K ratios in HD and control brains

**Fig. 2:**
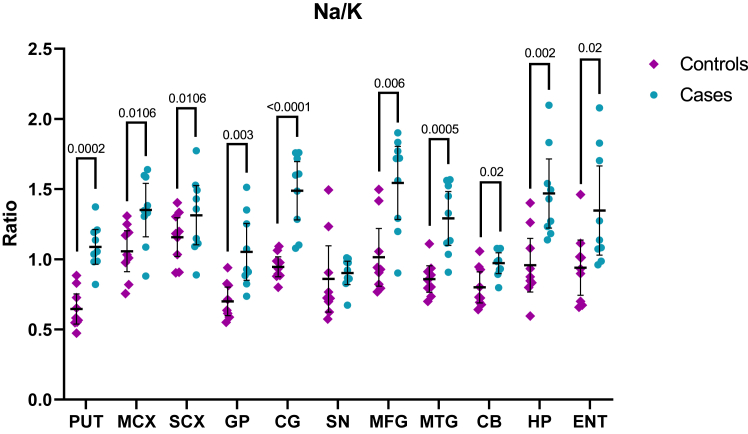
Each data point denotes the mean of three biological replicates; n = 7–9. Case–control differences determined by Mann–Whitney U test; p < 0.05 was considered significant. Purple diamonds represent controls and blue circles represent HD cases; error bars denote ±95% CI. C = Controls; CB = cerebellum; CG = Cingulate gyrus; ENT = Entorhinal cortex; GP = Globus pallidus; HD = HD cases; HP = Hippocampus; MCX = Motor cortex; MFG = Middle frontal gyrus; MTG = Middle temporal gyrus; PUT = Putamen; SCX = Sensory cortex; SN = Substantia nigra.

Na/K ratios were also determined in and compared between HD case and control brains to determine whether the relative concentrations of Na and K are altered in the HD brain; the ratios for each investigated brain region were as shown in Fig. 2 and Table 13.

**Table 13:** Na/K Ratios in HD and control brains.

	Controls	Cases	p value	S-value	E-value	Risk Ratio (RR)	RR CI E-values	Effect size
PUT	0.65 ± 0.14 (0.54–0.75)	1.09 ± 0.16 (0.96–1.21)	0.0002	12.3	8.5	4.5 (1.3–15.3)	1.9	3.2
MCX	1.06 ± 0.19 (0.91–1.20)	1.35 ± 0.25 (1.16–1.54)	0.0106	6.6	13.5	7.0 (1.1–45.9)	1.4	1.5
SCX	1.05 ± 0.18 (0.91–1.19)	1.48 ± 0.34 (1.21–1.74)	0.0106	6.6	7.5	4.0 (1.2–13.9)	1.7	2.4
GP	0.70 ± 0.13 (0.60–0.80)	1.05 ± 0.26 (0.85–1.26)	0.003	8.4	15.5	8.0 (1.2–51.5)	1.7	2.7
CG	0.95 ± 0.09 (0.87–1.02)	1.49 ± 0.27 (1.28–1.70)	<0.0001	13.3	8.5	4.5 (1.3–15.3)	1.9	5.8
SN	0.86 ± 0.31 (0.62–1.10)	0.90 ± 0.11 (0.82–0.99)	0.2	2.3	7.5	0.25 (0.01–3.7)	1.0	0.1
MFG	1.01 ± 0.27 (0.81–1.22)	1.54 ± 0.34 (1.28–1.80)	0.006	7.4	6.5	3.5 (1.0–12.5)	1.0	2.0
MTG	0.86 ± 0.12 (0.76–0.95)	1.29 ± 0.25 (1.10–1.48)	0.0005	11.0	15.5	8.0 (1.2–51.5)	1.7	3.5
CB	0.8 ± 0.14 (0.69–0.91)	0.97 ± 0.10 (0.90–1.05)	0.02	5.6	4.4	2.5 (0.6–9.7)	1.0	1.2
HP	0.96 ± 0.25 (0.77–1.15)	1.47 ± 0.32 (1.22–1.71)	0.002	9.0	4.4	2.5 (0.6–9.7)	1.0	2.1
ENT	0.94 ± 0.26 (0.74–1.14)	1.35 ± 0.41 (1.03–1.66)	0.02	5.6	7.5	4.0 (0.5–29.2)	1.0	1.6

Data shown are means ± SD (95% CI); n = 7–9. Case–control differences determined by Mann–Whitney U test; values in bold denote significant case–control differences; p < 0**.**05 was considered significant. CB = cerebellum; CG = Cingulate gyrus; CI = Confidence interval; ENT = Entorhinal cortex; GP = Globus pallidus; HP = Hippocampus; MCX = Motor cortex; MFG = Middle frontal gyrus; MTG = Middle temporal gyrus; PUT = Putamen; RR = risk ratio; SCX = Sensory cortex; SN = Substantia nigra.

Na/K ratios were found to be significantly increased across 10 out of 11 investigated brain regions, including the PUT (0.65 vs 1.09 in cases and controls, respectively; p = 0.0002; S-value = 12.3), MCX (1.06 vs 1.35; p = 0.0106; S-value = 7.0), SCX (1.05 vs 1.48; p = 0.0106; S-value = 6.6), GP (0.70 vs 1.05; p = 0.003; S-value = 8.4), CG (0.95 vs 1.49; p < 0.0001; S-value = 13.3), MFG (1.01 vs 1.54; p = 0.006; S-value = 7.4), MTG (0.86 vs 1.29; p = 0.0005; S-value = 11.0), CB (0.8 vs 0.97; p = 0.02; S-value = 5.6), HP (0.96 vs 1.47; p = 0.002; S-value = 9.0), and ENT (0.94 vs 1.35; p = 0.02; S-value = 5.6), with only the SN showing no significant changes (0.86 vs 0.90; p = 0.2). As such, even where significant alterations were not observed in Na or K individually, the Na/K ratio was often still significantly changed between HD cases and controls. Effect sizes were large in all regions except the SN (at 0.1), ranging from 1.2 in the CB to as high as 5.8 in the CG. Risk ratios were similarly large, ranging from 2.5 in the CB and HP to 8.0 in the GP; the risk ratio in the SN was 0.25.

### Individual profiles

Individual profiles were generated for each case and control based on each sample's average deviation from the control mean in terms of both individual metal concentrations across all investigated brain regions and concentrations of all nine measured analytes in individual regions. This was done to determine whether any individual samples overcontributed to any observed case–control differences; the generated profiles can be found in Supplementary Material Figure S3.

In terms of average deviation from control mean metal concentrations in individual regions, three cases and four controls showed a z-score >2: Case 1 in the HP, Case 4 in the GP, Case 8 in the ENT, Control 5 in the PUT, Control 7 in the MCX and MFG, and Control 1 in the CG. Following the exclusion of Case 1 in the HP, the p-value for case–control Na concentrations was no longer <0.05 (p = 0.0074; data not shown) and following the exclusion of Control 7 in the MFG, the p-value for case–control Ca concentrations = 0.09 (data not shown); as such, these findings require further investigation in an additional cohort. No other p-values moved across the 0.05 threshold following exclusion of these cases and controls.

In terms of average metal deviations across all 11 investigated regions, three cases and one control showed a z-score >2: Na levels in Case 9, Ca levels in Case 1, Mn levels in Case 2, and Mg and Ca levels in Control 1. Following the exclusion of Case 9, the p-value for Na case–control differences = 0.07 and following the exclusion of Case 1 and Control 1, the p-value for Ca levels in the MCX = 0.054; again, this indicates that further studies are required to determine the reliability of these findings. No other alterations were observed following the exclusion of these cases and controls.

## Discussion

In this study, a metallomic analysis of eight essential metals and Se was conducted in order to determine whether differences in the concentrations of these metals were present between HD cases and controls. The results revealed several metallic perturbations in the HD brain, including decreased Se in 11 out of 11 investigated regions and widespread increases in the Na/K ratio, as well as localised alterations in Ca, Zn, Cu, Mn, and Fe. Most of these alterations showed a risk ratio >3 and a moderate–high effect size, as well as moderate–good statistical significance, indicating the robustness of the observations despite the small sample size.

### Se

Se is an essential co-factor for many important metalloenzymes and proteins, including proliferent antioxidant enzymes such as glutathione peroxidases and thioredoxin reductases. As such, Se plays an important role in protection against oxidative stress and mitochondrial dysfunction—both of which are known to occur in the pathogenesis of HD.^13^ Such ubiquitous decreases in HD-brain Se as those observed in this study have not to our knowledge been reported elsewhere, although region-specific decreases have been observed in the primary visual cortex, the BA9 region of the frontal cortex, the CB, the CG, and the PUT; however, this study did not find decreases in the SN, MCX, or GP.^6^ It should be noted that this study investigated Se alterations in wet-weight tissues, whereas the current study used dry-weight tissue; wet weight analyses often produce greater variability due to varying water levels between samples, which may have contributed to these discrepancies. In the former study, liver decreases in Se in an HD mouse model were reversible with the administration of sodium selenite, as well as attenuation of motor deficits and neuronal loss. Similar results have been observed in another HD mouse model, in which co-supplementation with rutin and selenium prevented oxidative stress and inflammation caused by the administration of 3-nitropropionic acid (3-NPA).^14^ These changes resulting from early administration of Se (up to 14 weeks of age^6^) suggest early alterations in Se levels in these models. Together, these reports give robust evidence for the presence of diminished Se levels in the HD brain and also indicate that Se supplementation may be a viable therapeutic avenue for the treatment of HD.

However, caution must be exercised in this regard, as there is a tight window in which Se levels are neither too low nor too high, and incautious supplementation may lead to toxicity or harmful side-effects in humans; additionally, in contrast to brain tissue Se observations, plasma Se levels have been reported to be elevated in individuals with HD by as much as ∼40%^15^—Se supplementation may exacerbate such increases. This opposition between cerebral and blood Se levels may also indicate a disturbance of Se homeostasis across the blood–brain barrier (BBB), with BBB disruption and leaking having been observed in both an HD mouse model and human HD brains.^16^ Any Se-based therapeutic interventions would need to be able to circumvent BBB impairment without causing toxic accumulation of Se outside the brain; however, upon reaching the brain, the widespread presence of diminished Se might obviate the necessity of targeting specific brain regions. As such, potential Se-based therapies present with both strengths and difficulties for the treatment of HD. Se supplementation is already used with minimal side-effects to treat Se deficiency or its associated conditions; for example, Keshan disease—caused by dietary Se deficiency as a result of Se-depleted soil in some regions of China in combination with infection with a mutated strain of Coxsackievirus—can be both prevented^17^ and treated^18^ with oral Se supplementation with few to no harmful side-effects, supporting the safety profile of this treatment.

In terms of HD models, sodium selenite supplementation has been reported to be neuroprotective in the N171-82Q mouse model of HD, with decreased HTT aggregate burden and oxidative stress as well as altered selenoprotein transcription in the brain^6^. Importantly, although these HD model mice also show mildly elevated plasma levels of Se, supplementation with sodium selenite did not exacerbate this; this suggests that supplementation with this form of Se may be safe despite the elevated plasma Se levels observed in individuals with HD.^15^ Coadministration of selenium nitrate with rutin was found to have similar effects in the 3-NPA HD mouse model brain, although peripheral effects were not investigated.^14^ In a *C. elegans* model of HD, SeNPs were able to prevent the aggregation of HTT and decrease oxidative stress at low doses below 2 μM^19^; such SeNPs may present an even safer form of Se supplementation that would also allow for a more controlled release of Se time, avoiding Se spikes and troughs.^20^ Considering the easy availability and the observed safety of sodium selenite supplementation in animal models, this may be a candidate for future trials with human subjects—although administration of SeNPs may present a safer, more controllable alternative should supplementation with sodium selenite be found to cause dangerous elevations in peripheral Se levels.

Despite the findings of animal studies, the results from the current human brain study should be treated as preliminary due to the size of the cohort employed; further investigations should first confirm the Se observations observed here in a larger, more diverse cohort. Mouse models have indicated potential mechanisms of Se deficiency such as increased antioxidation^6^ and decreased inflammatory activity and apoptosis,^14^ which have also been observed in human HD brains^13^^,^^21^^,^^22^ n. It would be of great utility to try to determine at what point these harmful processes and Se alterations occur in the human HD brain, either through investigations of pre-clinical post-mortem HD brain tissues (the genetic nature of the disease makes this possible in theory, although practical difficulties in obtaining sufficient samples for such a study may preclude this in practice) or by investigating potential Se-related alterations in the living human brain via imaging. For example, reduced levels of glutathione (an important antioxidative selenoprotein) have been observed in the post-mortem HD caudate nucleus,^23^ inferior temporal cortex, frontal pole, and middle temporal cortex,^24^ but this has not been investigated in vivo in HD using the available imaging techniques^25^; an investigation of this analyte at preclinical stages may support the presence early-stage Se-related oxidative stress. Unfortunately, Se levels themselves cannot be measured in the living human brain at present with existing techniques, and so only post-mortem measurements are possible.

On an additional note, it should be noted that although Se levels have also been observed to be relatively low in some areas of New Zealand (where the current study cohort was derived from), there is little evidence for sufficient plasma Se decreases in the general population to result in clinical consequences^26^; furthermore, both case and control tissues were obtained from Auckland Brain Bank from New Zealand-based donors, and so any effects of any such soil depletion should not be able to account for the case–control observations in the current cohort.

### Na and K

Na and K ions are crucial for cellular homeostasis—playing a particularly important role in the central nervous system by regulating the generation of action potentials and the subsequent conduction of electrical nerve impulses via Na^+^–K^+^ ATPase (AKA the Na^+^/K^+^ pump). Although the Na/K ratio itself has not—to the authors’ knowledge—been previously investigated in HD, widespread Na increases correlated with grey matter loss were reported in a study by Reetz et al.,^5^ including in regions such as the PUT, CG, and HP—as observed here. Interestingly, Na alterations were already present in two preclinical HD cases in the Reetz et al. study, albeit only in the thalamus, with Na changes in the PUT and HP being present by early clinical stages of the disease. Another study by Gramsbergen et al.^27^ also identified Na increases and K decreases in the HD SN and PUT. As such, perturbations in Na and K levels, and by extension the Na/K ratio, appear to be a feature of early clinical disease in HD.

Hypernatraemia (elevated Na levels in the blood) has not been reported as a common event in HD to the authors’ knowledge, nor has hypokalaemia (deficient K). Dehydration may lead to hypernatraemia in elderly individuals, but in the current study, we observed no differences in case–control brain tissue water content (see Supplementary Material B for all tissue wet and dry weights). Instead, the observed alterations in the Na/K ratio may reflect Na^+^/K^+^ pump dysfunction in the brain, resulting in a loss of Na/K homeostasis. Unfortunately, studies of Na^+^–K^+^ ATPase in whole human HD brain tissues are lacking, but increased Na^+^–K^+^ ATPase levels have been observed in HD erythrocytes.^28^^,^^29^ The Na/K pump requires a substantial amount of energy, using up to a fifth of available cellular ATP^30^; a decrease in energy production, e.g., via mitochondrial dysfunction or reduced glucose metabolism—as seen in HD^31^^,^^32^—may cause insufficient ATP to be available for the maintenance of Na/K homeostasis. Studies of Na^+^–K^+^ ATPase activity in the HD brain may, therefore, be able to provide a link between the increased glucose levels^33^ and glucose hypometabolism, increased oxidative stress, and Na/K dyshomeostasis observed in the HD brain.

### Ca

Ca is a major intracellular messenger that plays many roles within the brain—most predominantly in neuronal signalling and neurotransmitter release. As a result, disruptions in intra/extracellular Ca balance may result in the dysfunction of several cellular processes, including mitochondrial oxidative phosphorylation, protein folding, transcriptional regulation, and protein synthesis. Ca increases in particular—as seen in several regions of the HD brain in the current study—may reflect hyperexcitotoxicity in the brain, in which overactivation of glutamate receptors leads to excessive Ca influx, which may result in mitochondrial Ca toxicity and the activation of degradative enzymes over time. Isolated human and mouse model HD mitochondria have been reported to be more sensitive to Ca^2+^ loads, depolarising at lower Ca^2+^ concentrations^34^—with the expression of mHTT being sufficient to decrease the Ca^2+^ threshold for mitochondrial permeability transition pore (MPT) opening in mouse liver mitochondria.^35^ Opening of the MPT can lead to further Ca influx, mitochondrial swelling, and cell death. Notably, striatal mitochondria appear to show higher Ca^2+^ sensitivity than mitochondria isolated from cortical neurons in HD mouse models, although they also appear to develop resistance over time.^36^ As such, Ca levels may be another promising target for intervention in HD, although they appear to be disturbed in a more localised and temporal manner than Se, which may make the appropriate brain region and disease stage-specific delivery of potential therapeutics more difficult.

### Zn, Mn, Fe, and Cu

Like Se, Zn, Mn, Fe, and Cu are important metal cofactors for a great number of enzymes, including some that are involved in antioxidation—e.g., superoxide dismutase (SOD)—and others involved in processes as wide-ranging as gene expression, cell signalling, and apoptosis. As such, perturbations in these metals may affect a wide range of essential cellular processes. Deficiencies in Mn and Cu may lead to reduced antioxidative ability, leading to mitochondrial dysfunction.^37^^,^^38^ Although Zn is also a cofactor for some antioxidative enzymes, excess Zn can itself result in mitochondrial oxidative stress by disrupting the functioning of the ETC, e.g., by reducing NAD ^+^ levels.^39^ Excess Zn can also contribute to dysfunctional Ca homeostasis (as was observed in the current study) via excitotoxic glutamatergic signalling^40^; as such, alterations in Ca and Zn may be closely linked. Likewise, Mn, Cu, and Se deficiency and excess Zn may each contribute to a cumulative state of oxidative stress within the HD brain, with some brain regions being more susceptible to alterations in individual elements. Decreased Fe levels can lead to cognitive impairment^41^ and motor dysfunction,^42^ with the striatal dopaminergic system appearing to be particularly vulnerable to Fe deficiency.^41^ Although Fe overload is often associated with oxidative stress via the production of reactive oxygen species in the Fenton reaction, Fe deficiency can also damage mitochondria and lead to oxidative stress.^42^

Several studies have reported increased Fe levels in the HD brain,^4^^,^^8^, ^9^, ^10^ with only a single report observing decreases in the frontal lobe and genu white matter^8^ and one in the corpus callosum^7^; two studies have reported increases in the GP—the only region in which decreased Fe was observed in the current study.^8^^,^^10^ Increases in HD PUT Fe have also been reported^10^; statistically significant increases were not observed here, although there was an upwards trend in the PUT. The discrepancies between our current observations and previous imaging studies may reflect differences in disease staging and methodologies; although such discrepancies are present, it is important to realise the potential of imaging metals in living individuals in the HD brain that is presented by existing Fe MRI methodologies. Methods have been developed by which Na and K can be measured in the brain using MRI, and so studies of the development of Na/K ratio changes from the preclinical stage of HD throughout the clinical stages may be feasible and of great use in determining the temporality of such changes; in particular, preclinical or early-stage alterations would suggest a mechanistic role for these changes in HD, rather than merely being the result of other pathogenic mechanisms. The development of similar methods for other metals such as Se would also be of great utility. Further Fe imaging studies assessing the development of Fe alterations from early to late stages of HD would also be of interest to assess whether Fe patterns change as symptoms progress; at this moment in time, most studies have focused on only early or late-stage HD or have not assessed the same individuals at different time points to determine the temporality of alterations alongside clinical progression.

Zn has previously been observed to be increased in the HD pallidum and putamen,^4^ as well as in the blood of HD patients.^15^ Decreased Cu has also been previously observed in the HD anterior CG and decreased Mn in the anterior CG and superior frontal gyrus by the same group.^4^ Alterations in these metals have also been observed in HD patient CSF; elevations in CSF Mn and Zn levels appear to manifest prior to the development of HD biomarkers, whilst Fe increases are present in clinically manifest patients.^43^ Such observations indicate changes in CSF metal concentrations prior to the development of clinical symptoms or mHTT aggregation in individuals with HD, suggesting mechanistic roles for these metals.

Some of these metal alterations have also been observed in HD animal model brains. For example, decreased Mn levels have been observed in YAC128 HD mouse model brains, with resultant motor, behavioural, and cognitive changes; these changes cannot be attenuated with chronic Mn exposure, but it does appear to increase dopamine release in YAC128 mice without the negative effects seen in wildtype mice—showing differing metabolic, transcriptional, and protein responses to Mn exposure.^44^, ^45^, ^46^ Likewise, the n171-82QHD mouse model has shown increased Fe levels alongside concurrent microglial morphological alterations indicative of immune activation as well as neurodegeneration with further neonatal Fe supplementation.^47^ In addition, Zn deficiency has been observed in the HP and cortex of the R6/1 mouse model of HD, with dietary Zn restriction exacerbating HP long-term potentiation deficits, AMPA receptor decreases, and cognitive impairment in this model.^48^

As such, our observations concerning Zn increases in the GP and MFG, decreased Mn in the SN, and decreased Cu in the CB have not been reported previously in human HD brains, whereas there is some support in the existing literature for the Zn increases observed in the PUT and some discrepancies concerning our Fe observations. Animal HD model studies appear to support our findings in Mn and Fe, with mouse models showing similar changes to those observed here. However, mouse models of HD have been reported to show Zn deficiency in the cortex and HP, in contrast to the lack of change observed in the HP here and the increases found in the GP and MFG; considering the Zn increases reported by Rosas et al. in the brains of humans with HD, this may represent a discrepancy in cerebral Zn changes between human HD brains and HD mouse models.

### Limitations

There were several limitations to this study. Foremost was the small sample size, which could have resulted in more subtle significant metal alterations being missed. Due to the limited and precious nature of brain samples, this limitation is difficult to overcome—particularly in studies of multiple brain regions; however, despite this, we were able to observe many alterations with sizeable effect sizes. The selection of this sample size was based on previous metallomic studies of Alzheimer's and Parkinson's disease dementia brains in which significant case–control alterations were observable using cohorts of this number.^49^^,^^50^ As such, we believe that this sample set can serve as a starting point for the investigation of the identified metals of interest, such as Se, Na, and K. Further studies employing larger sample sizes would now strengthen these findings.

Another limitation of this study is the lack of data available on the samples; all available data has been supplied here, but there was unfortunately no information on comorbidities, nutritional status, body-mass index, etc., available for these samples. As such, there may be additional differences between cases and controls that are not accounted for here. Where possible, the samples have been matched—such as for age, sex, and PMD; however, almost all cases had bronchopneumonia listed as their cause of death, whereas controls mostly died from ischaemic heart disease. The differences in causes of death between cases and controls may have contributed to the findings observed here. In the case of Se, this seems unlikely, as decreased Se levels are often associated with heart disease^51^; as such, we would have expected to have found decreased Se in controls rather than in HD cases if this were the case. However, it remains possible that these differences contributed to alterations in other metals, despite the causes of death primarily deriving from non-CNS organs. It is also possible that the small (although non-significant) difference in age between cases and controls may have affected their respective metal concentrations; relationships between age and metal levels could be investigated in a larger cohort.

### Conclusions

Taken together, the metallomic disturbances observed in this study indicate metallic contributions to a state of oxidative stress and perturbed energy balance via mitochondrial dysfunction in the HD brain caused by deficiencies in Se, Cu, Fe, and Mn and increased Zn, as well as disturbances in cellular homeostasis and neuronal signalling via dyshomeostasis of Na and K levels. Se deficiency presents a promising therapeutic target due to its presence in 11 out of 11 investigated brain regions, with mouse model studies showing promising findings. The development of a safe and effective drug targeting Se deficiency in the HD brain should be investigated further.

## Contributors

MS: Conceptualisation, data curation, formal analysis, investigation, methodology, project administration, validation, visualisation, writing—original draft, review, and editing. SP: Investigation and methodology. JX: Investigation and methodology. GJSC: Conceptualisation, funding acquisition, resources, supervision, writing—review and editing. MS and GJSC verified the underlying data for this manuscript. All authors read and approved the final version of the manuscript.

## Data sharing statement

All raw data for individual runs, as well as mean values of triplicate runs, are included in the Supplementary Materials; this also includes blank values, standard curve values, tissue wet and dry weights, and indication of if values were excluded from analysis as outliers or due to high blank values. Concentrations are given in both mmol per kg/nmol per kg and ug/l. Values are given for mean averages, standard deviations, confidence intervals, and Mann–Whitney U test results. Details of all software used for analyses is included in the methods section.

## Declaration of interests

The authors are solely responsible for the decision to publish these data and are solely responsible for its content and the writing of the article. A patent disclosing selenium treatment for Huntington's disease has been submitted to the US Patent and Trade Office for examination by MS and GJSC; the patent will be owned by the University of Manchester and the authors have no duality of interest. MS has received travel support from Alzheimer's Research UK, Guarantors of Brain, and the British Neuroscience Association. JX and SP declare that they have nothing to disclose.
