# Supplementary material for: Widespread selenium deficiency in the brain of cases with Huntington's disease presents a new potential therapeutic target
Source: eBioMedicine. 2023 Oct 10;97:104824. doi: 10.1016/j.ebiom.2023.104824 (PMC10667115; doi:10.1016/j.ebiom.2023.104824)
Supplement: Supplementary Materials A [file mmc1.docx]

# Supplementary Material A

Contents

[Supplementary Material A 1](#_Toc132900457)

[Supplementary Table 1. Individual Donor Characteristics 2](#_Toc132900458)

[Supplementary Table 2A. ICP–MS Analysis of HD and Control PUT 4](#_Toc132900459)

[Supplementary Table 2B. ICP–MS Analysis of HD and Control MCX 4](#_Toc132900460)

[Supplementary Table 2C. ICP–MS Analysis of HD and Control SCX 5](#_Toc132900461)

[Supplementary Table 2D. ICP–MS Analysis of HD and Control GP 5](#_Toc132900462)

[Supplementary Table 2E. ICP–MS Analysis of HD and Control CG 6](#_Toc132900463)

[Supplementary Table 2F. ICP–MS Analysis of HD and Control SN 6](#_Toc132900464)

[Supplementary Table 2G. ICP–MS Analysis of HD and Control MFG 7](#_Toc132900465)

[Supplementary Table 2H. ICP–MS Analysis of HD and Control MTG 7](#_Toc132900466)

[Supplementary Table 2I. ICP–MS Analysis of HD and Control CB 8](#_Toc132900467)

[Supplementary Table 2J. ICP–MS Analysis of HD and Control HP 8](#_Toc132900468)

[Supplementary Table 2K. ICP–MS Analysis of HD and Control ENT 9](#_Toc132900469)

[Supplementary Table 2L. ICP–MS Analysis of HD and Control Na/K Ratios 9](#_Toc132900470)

[Supplementary Discussion – Case and Control Metallomic Regional Variations 10](#_Toc132900471)

[Na 10](#_Toc132900472)

[Mg 10](#_Toc132900473)

[K 10](#_Toc132900474)

[Ca 11](#_Toc132900475)

[Mn 11](#_Toc132900476)

[Fe 12](#_Toc132900477)

[Cu 12](#_Toc132900478)

[Zn 12](#_Toc132900479)

[Se 13](#_Toc132900480)

[Supplementary Figure 1: Metal Concentrations Across Control Brain Regions 14](#_Toc132900481)

[Supplementary Figure 2: Metal Concentrations Across HD Case Brain Regions 15](#_Toc132900482)

[Supplementary Discussion—Application of Bradford-Hill Criteria to Se Findings 16](#_Toc132900483)

[Supplementary Figure 3: Individual Sample Profiles 18](#_Toc132900484)

## Supplementary Table 1. Individual Donor Characteristics

| Sample | Status | Age (years) | Sex | PMD (hours) | Brain weight (g) | CAG repeats | CAP Score | Vonsattel Grade | Cause of Death |
| --- | --- | --- | --- | --- | --- | --- | --- | --- | --- |
| H01 | HD | 80 | M | 9 | 1105 | 20/40 | 123.3 | 1 | Bronchopneumonia |
| H02 | C | 63 | M | 9 | 1432 | 19/20 | - | - | Ischaemic Heart Disease |
| H03 | HD | 67 | F | 9 | 1139 | 15/42 | 123.9 | 1 | Myocardial Infarction |
| H04 | C | 61 | M | 7 | 1258 | 17/19 | - | - | Ischaemic Heart Disease |
| H05 | C | 77 | F | 12 | 1227 | 18/20 | - | - | Coronary Atherosclerosis |
| H06 | HD | 59 | F | 7 | 787 | 23/47 | 154.5 | 4 | Bronchopneumonia |
| H07 | C | 73 | F | 13 | 1210 | 17/17 | - | - | Ischaemic Heart Disease |
| H08 | HD | 62 | F | 11 | 826 | 17/45 | 143.3 | 3 | Bronchopneumonia |
| H09 | C | 73 | M | 13 | 1315 | 17/23 | - | - | Ischaemic Heart Disease |
| H10 | HD | 62 | M | 9 | 992 | 18/43 | 124.2 | 2 | Huntington's disease |
| H11 | C | 49 | M | 13 | 1495 | 17/17 | - | - | Ischaemic Heart Disease |
| H12 | HD | 83 | M | 13 | 1168 | 17/42 | 153.5 | 1 | Bronchopneumonia |
| H13 | C | 66 | M | 15 | 1360 | 15/20 | - | - | Ischaemic Heart Disease |
| H14 | HD | 58 | M | 14 | 1497 | 28/44 | 125.1 | 2 | Bronchopneumonia |
| H15 | C | 64 | F | 6.5 | 1260 | 18/23 | - | - | Pulmonary Embolism |
| H16 | HD | 51 | M | 15 | 1200 | 10/46 | 125.7 | 2 | Bronchopneumonia |
| H17 | C | 81 | M | 7 | 1343 | 15/18 | - | - | Coronary Atherosclerosis |
| H18 | HD | 65 | M | 14 | 1224 | 17/43 | 130.2 | 2 | Renal Failure |

All HD samples were confirmed to have the disease with genetic testing, with all having >40 CAG repeats. The most common cause of death in control samples was ischaemic heart disease, whereas bronchopneumonia was the most common cause in HD donors.

### Supplementary Table 2A. ICP–MS Analysis of HD and Control PUT

| **PUT** | **Controls** | **Cases** | **p–value** |
| --- | --- | --- | --- |
| Na | 250·7  (209·7–291·6) | 362·9  (325·4–400·4) | 0·0003 |
| Mg | 26·1  (25·0–27·3) | 25·3  (23·8–26·7) | 0·3 |
| K | 388·7  (373·3–404·0) | 334·6  (320·1–349·2) | 0·00002 |
| Ca | 5·7  (4·9–6·5) | 6·7  (6·3–7·1) | 0·02 |
| Mn | 45·7  (38·0–53·4) | 40·6  (30·5–50·6) | 0·4 |
| Fe | 14·2  (9·7–18·7) | 16·8  (10·7–22·9) | 0·4 |
| Cu | 520·3  (397·5–643·1) | 455·0  (376·0–534·1) | 0·3 |
| Zn | 1016·1  (938·7–1093·5) | 1261·3  (1103·5–1419·1) | 0·008 |
| Se | 14·6  (12·9–16·2) | 10·6  (9·8–11·3) | 0·0004 |

### Supplementary Table 2B. ICP–MS Analysis of HD and Control MCX

| **MCX** | **Controls** | **Cases** | **p–value** |
| --- | --- | --- | --- |
| Na | 299·4  (259·6–339·1) | 362·3  (317·1–407·5) | 0·03 |
| Mg | 22·0  (20·7–33·3) | 22·1  (21·0–23·2) | 0·9 |
| K | 285·5  (260·0–311·0) | 271·3  (246·5–296·1) | 0·4 |
| Ca | 5·6  (5·1–6·1) | 6·7  (6·0–7·5) | 0·02 |
| Mn | 17·8  (15·2–20·3) | 21·3  (17·7–24·9) | 0·2 |
| Fe | 5·1  (4·4–5·7) | 5·0  (4·3–5·8) | 0·9 |
| Cu | 319·8  (233·6–406·0) | 324·6  (269·9–379·3) | 0·9 |
| Zn | 808·4  (724·6–892·2) | 897·3  (802·8–991·8) | 0·1 |
| Se | 11·1  (9·2–13·0) | 9·0  (7·9–10·0) | 0·1 |

### Supplementary Table 2C. ICP–MS Analysis of HD and Control SCX

| **SCX** | **Controls** | **Cases** | **p–value** |
| --- | --- | --- | --- |
| Na | 335·7  (295·6–375·8) | 404·1  (367·0–441·2) | 0.011 |
| Mg | 24·1  (22·8–25·5) | 24·1  (22·5–25·7) | 1.0 |
| K | 322·6  (294·0–351·3) | 282·0  (243·3–320·7) | 0.1 |
| Ca | 5·7  (5·0–6·4) | 6·2  (5·2–7·2) | 0.3 |
| Mn | 20·0  (18·2–21·7) | 19·9  (17·1–22·8) | 1.0 |
| Fe | 5·4  (4·8–6·0) | 5·1  (4·6–5·5) | 0.4 |
| Cu | 360·4  (266·4–454·3) | 295·9  (265·8–326·1) | 0.2 |
| Zn | 913·8  (839·4–988·2) | 967·4  (859·4–1075·3) | 0.4 |
| Se | 12·5  (11·2–13·8) | 10·1  (9·0–11·2) | 0.1 |

### Supplementary Table 2D. ICP–MS Analysis of HD and Control GP

| **GP** | **Controls** | **Cases** | **p–value** |
| --- | --- | --- | --- |
| Na | 197·0  (167·1–226·9) | 271·4  (218·6–324·3) | 0.015 |
| Mg | 21·0  (19·6–22·3) | 21·5  (20·2–22·8) | 0.6 |
| K | 282·6  (259·1–306·2) | 258·3  (248·4–268·3) | 0.051 |
| Ca | 6·1  (4·4–7·9) | 5·5  (4·8–6·2) | 0.5 |
| Mn | 33·0  (29·0–37·0) | 30·0  (24·4–35·6) | 0.3 |
| Fe | 12·6  (9·4–15·8) | 8·7  (5·2–12·2) | 0.1 |
| Cu | 344·3  (287·9–400·6) | 312·8  (249·7–375·9) | 0.4 |
| Zn | 749·0  (692·8–805·2) | 878·4  (796·7–960·1) | 0.009 |
| Se | 11·8  (10·3–13·2) | 8·8  (8·1–9·5) | 0.0014 |

### Supplementary Table 2E. ICP–MS Analysis of HD and Control CG

| **CG** | **Controls** | **Cases** | **p–value** |
| --- | --- | --- | --- |
| Na | 360·7  (308·9–412·5) | 520·7  (458·4–583·0) | 0.0004 |
| Mg | 27·4  (24·7–30·1) | 28·3  (26·9–29·7) | 0.5 |
| K | 401·8  (375·7–428·0) | 352·1  (334·4–369·9) | 0.2 |
| Ca | 6·9  (5·8–8·0) | 9·6  (7·3–12·0) | 0.04 |
| Mn | 22·5  (19·5–25·5) | 22·6  (20·3–25·0) | 0.9 |
| Fe | 4·3  (3·7–4·9) | 4·5  (3·9–5·2) | 0.5 |
| Cu | 370·0  (252·4–487·6) | 372·1  (314·4–429·7) | 1.0 |
| Zn | 1052·3  (843·0–1261·7) | 1247·5  (1136·9–1358·2) | 0.1 |
| Se | 13·4  (10·2–13·6) | 10·6  (9·8–11·3) | 0.1 |

### Supplementary Table 2F. ICP–MS Analysis of HD and Control SN

| **SN** | **Controls** | **Cases** | **p–value** |
| --- | --- | --- | --- |
| Na | 238·5  (184·2–292·8) | 222·0  (207·6–236·4) | 0.5 |
| Mg | 21·4  (20·1–22·7) | 20·3  (19·4–21·1) | 0.1 |
| K | 282·4  (260·8–304·0) | 247·7  (233·0–262·5) | 0.008 |
| Ca | 5·5  (3·8–7·3) | 4·6  (4·3–4·9) | 0.3 |
| Mn | 25·2  (21·2–29·2) | 19·8  (16·8–22·8) | 0.02 |
| Fe | 8·8  (5·4–12·2) | 6·1  (3·5–8·6) | 0.2 |
| Cu | 579·5  (365·3–793·6) | 361·9  (174·4–549·3) | 0.13 |
| Zn | 846·7  (714·2–979·3) | 761·0  (625·1–897·0) | 0.3 |
| Se | 14·2  (10·3–18·1) | 8·7  (7·9–9·5) | 0.01 |

### Supplementary Table 2G. ICP–MS Analysis of HD and Control MFG

| **MFG** | **Controls** | **Cases** | **p–value** |
| --- | --- | --- | --- |
| Na | 337·6  (280·4–394·8) | 462·0  (406·3–517·8) | 0.002 |
| Mg | 24·0  (23·2–24·8) | 24·9  (23·6–26·2) | 0.2 |
| K | 337·0  (311·9–362·1) | 307·5  (265·3–349·7) | 0.2 |
| Ca | 5·8  (5·0–6·6) | 6·7  (6·2–7·3) | 0.1 |
| Mn | 19·5  (18·3–20·6) | 21·6  (17·9–25·3) | 0.2 |
| Fe | 4·8  (4·2–5·3) | 5·1  (4·5–5·7) | 0.4 |
| Cu | 347·9  (283·6–412·1) | 337·0  (281·3–392·8) | 0.8 |
| Zn | 958·9  (883·7–1034·1) | 1111·8  (973·4–1250·2) | 0.04 |
| Se | 12·8  (11·1–14·4) | 10·6  (9·4–11·7) | 0.1 |

### Supplementary Table 2H. ICP–MS Analysis of HD and Control MTG

| **MTG** | **Controls** | **Cases** | **p–value** |
| --- | --- | --- | --- |
| Na | 352·6  (303·1–402·0) | 498·9  (432·1–565·7) | 0.0011 |
| Mg | 28·5  (26·9–30·0) | 29·5  (27·9–31·1) | 0.3 |
| K | 410·9  (374·2–447·7) | 390·3  (352·6–428·1) | 0.4 |
| Ca | 6·3  (5·4–7·2) | 8·1  (7·2–9·1) | 0.006 |
| Mn | 19·6  (17·8–21·5) | 21·0  (18·0–24·0) | 0.4 |
| Fe | 5·6  (5·0–6·2) | 5·3  (4·8–5·8) | 0.3 |
| Cu | 416·2  (312·0–520·4) | 377·2  (311·6–442·8) | 0.5 |
| Zn | 1119·7  (973·5–1265·9) | 1304·0  (1161·5–1446·6) | 0.054 |
| Se | 17·0  (12·5–21·4) | 11·8  (10·6–12·9) | 0.03 |

### Supplementary Table 2I. ICP–MS Analysis of HD and Control CB

| **CB** | **Controls** | **Cases** | **p–value** |
| --- | --- | --- | --- |
| Na | 369·6  (307·7–431·4) | 405·3  (376·2–434·3) | 0.3 |
| Mg | 31·4  (29·3–33·5) | 29·9  (28·6–31·1) | 0.2 |
| K | 460·9  (427·1–494·7) | 417·9  (395·9–440·0) | 0.03 |
| Ca | 7·0  (6·0–7·9) | 6·7  (5·8–7·6) | 0.6 |
| Mn | 37·9  (34·6–41·2) | 36·5  (30·8–42·3) | 0.7 |
| Fe | 5·4  (4·2–6·6) | 5·2  (4·1–6·3) | 0.8 |
| Cu | 648·3  (533·8–762·7) | 492·3  (414·9–569·8) | 0.02 |
| Zn | 1236·9  (1107·3–1366·5) | 1118·6  (1017·6–1219·6) | 0.1 |
| Se | 28·2  (11·2–45·3) | 18·0  (11·8–24·3) | 0.06 |

### Supplementary Table 2J. ICP–MS Analysis of HD and Control HP

| **HP** | **Controls** | **Cases** | **p–value** |
| --- | --- | --- | --- |
| Na | 351·9  (309·6–394·1) | 437·6  (377·4–497·7) | 0.02 |
| Mg | 28·8  (26·5–31·1) | 26·9  (25·4–28·3) | 0.1 |
| K | 378·1  (333·0–423·2) | 301·7  (272·1–330·4) | 0.006 |
| Ca | 6·7  (5·8–7·5) | 10·3  (3·8–16·9) | 0.2 |
| Mn | 25·6  (19·6–31·6) | 24·3  (19·6–28·9) | 0.7 |
| Fe | 3·8  (3·4–4·3) | 3·6  (3·1–4·1) | 0.3 |
| Cu | 286·0  (240·3–331·8) | 256·1  (225·7–286·5) | 0.2 |
| Zn | 1310·1  (1119·5–1500·6) | 1208·6  (1057·3–1359·9) | 0.4 |
| Se | 15·6  (11·2–20·0) | 9·6  (8·0–11·2) | 0.015 |

### Supplementary Table 2K. ICP–MS Analysis of HD and Control ENT

| **ENT** | **Controls** | **Cases** | **p–value** |
| --- | --- | --- | --- |
| Na | 326·3  (270·5–382·1) | 422·7  (384·2–461·1) | 0.005 |
| Mg | 25·7  (24·4–27·1) | 25·8  (23·8–27·7) | 1.0 |
| K | 351·8  (321·3–382·4) | 329·7  (280·6–378·8) | 0.4 |
| Ca | 6·5  (5·5–7·6) | 7·3  (6·4–8·1) | 0.2 |
| Mn | 20·9  (18·7–23·1) | 20·8  (18·1–23·5) | 1.0 |
| Fe | 4·4  (3·7–5·1) | 4·4  (3·9–4·9) | 0.9 |
| Cu | 327·8  (257·8–398·2) | 316·1  (258·4–373·9) | 0.8 |
| Zn | 1095·4  (989·5–1201·3) | 1169·2  (1058·5–1279·9) | 0.3 |
| Se | 13·9  (11·3–16·5) | 9·7  (8·9–10·5) | 0.006 |

### Supplementary Table 2L. ICP–MS Analysis of HD and Control Na/K Ratios

| **Na/K** | **Controls** | **Cases** | **p–value** |
| --- | --- | --- | --- |
| PUT | 0·65 (0·54–0·75) | 1·09 (0·96–1·21) | 0.000013 |
| MCX | 1·06 (0·91–1·20) | 1·35 (1·16–1·54) | 0.013 |
| SCX | 1·05 (0·91–1·19) | 1·48 (1·21–1·74) | 0.006 |
| GP | 0·70 (0·60–0·80) | 1·05 (0·85–1·26) | 0.004 |
| CG | 0·95 (0·87–1·02) | 1·49 (1·28–1·70) | 0.0002 |
| SN | 0·86 (0·62–1·10) | 0·90 (0·82–0·99) | 0.7 |
| MFG | 1·01 (0·81–1·22) | 1·54 (1·28–1·80) | 0.002 |
| MTG | 0·86 (0·76–0·95) | 1·29 (1·10–1·48) | 0.0006 |
| CB | 0·8 (0·69–0·91) | 0·97 (0·90–1·05) | 0.0098 |
| HP | 0·96 (0·77–1·15) | 1·47 (1·22–1·71) | 0.002 |
| ENT | 0·94 (0·74–1·14) | 1·35 (1·03–1·66) | 0.03 |

Table 1A–L. Data shown are means (95% confidence intervals). P-values determined by Welch’s t-test.

## Supplementary Discussion – Case and Control Metallomic Regional Variations

### Na

In controls, Na levels were lowest in the GP, being significantly lower than in the CG (p = 0.0005; see Supplementary Figure 2), MFG (p = 0.04), MTG (p = 0.004), CB (396.6 mmol/kg dry weight; p = 0.002), and HP (p = 0.004). Control Na levels were highest in the CG, although they were only significantly higher than in the SN (238.5 mmol/kg dry weight; p = 0.04).

In cases, Na levels were lowest in the SN, being significantly lower than in the MFG (p = 0.0005; see Supplementary Figure 3), MTG (p < 0.0001), CB (405.3 mmol/kg dry weight; p = 0.02), HP (p = 0.004), and ENT (p 0.007). GP Na levels were similarly low, being significantly lower than in the CG (p < 0.0001), MFG (p = 0.0104), and MTG (p = 0.0006).

### Mg

In controls, Mg levels were lowest in the MCX (22.0 mmol/kg dry weight), SCX (24.1 mmol/kg dry weight), and GP (21.0 mmol/kg dry weight), being significantly lower in comparison to the CG (27.4 mmol/kg dry weight; p = 0.005; see Supplementary Figure 2), MTG (28.5 mmol/kg dry weight; p = 0.004), CB (31.4 mmol/kg dry weight; p < 0.0001), and HP (28.8 mmol/kg dry weight; p = 0.005). Mg concentrations were also lower in the control SN (21.4 mmol/kg dry weight) compared to the MTG (p = 0.0011), CB (p < 0.0001), and HP (p = 0.0014).

Mg concentrations varied considerably across different HD case regions; the lowest were observed in the SN (20.3 mmol/kg dry weight), being significantly lower than those seen in the CG (28.3 mmol/kg dry weight; p < 0.0001; see Supplementary Figure 3), MTG (29.5 mmol/kg dry weight; p < 0.0001), CB (29.9 mmol/kg dry weight; p < 0.0001), HP (26.9 mmol/kg dry weight; p = 0.0027), and ENT (25.8 mmol/kg dry weight; p = 0.004). The GP showed the second lowest concentrations (21.5 mmol/kg dry weight), with significant differences compared to the CG (p = 0.0015), MTG (p < 0.0001), CB (p < 0.0001), and HP (p = 0.0453). The MCX (22.1 mmol/kg dry weight) also showed lower Mg levels in comparison to the CG (p = 0.009), MTG (p = 0.0007), and CB (p = 0.0002). The highest concentrations were present in the MTG and CB.

### K

In controls, K levels varied across brain regions. The highest levels were observed in the CB (460.9 mmol/kg dry weight), being significantly higher than in the MCX (285.5 mmol/kg dry weight; p < 0.0001; see Supplementary Figure 2), SCX (p < 0.0001), GP (258.3 mmol/kg dry weight; p < 0.0001), and SN (p < 0.0001). In comparison to these four regions, K levels were also significantly higher in the PUT (p = 0.03, 0.04, 0.02, and 0.03, respectively), CG (p = 0.02, 0.02, 0.008, and 0.0103, respectively), and MTG (p = 0.003, 0.004, 0.002, and 0.002, respectively).

In cases, K levels were similarly variant across different brain regions. The highest levels were again observed in the CB (417.9 mmol/kg dry weight), being significantly higher than in the MCX (271.3 mmol/kg dry weight; p = 0.0003; see Supplementary Figure 3), SCX (p = 0.002), GP (258.3 mmol/kg dry weight; p < 0.0001), and SN (p < 0.0001)—as seen in the controls. Similarly, K levels in the MTG (390.3 mmol/kg dry weight) were also significantly higher than in these four regions in HD cases (p = 0.0054, 0.03, 0.0005, and < 0.0001, respectively).

### Ca

Ca levels in controls were very consistent, with no significant differences between any two regions (see Supplementary Figure 2). Ca levels were also fairly consistent across HD case brain regions, although they were somewhat lower in the SN (4.6 mmol/kg dry weight), being significantly lower compared to the CG (p < 0.0001; see Supplementary Figure 3), MTG (p < 0.0001), HP (10.3 mmol/kg dry weight; 0.007), and ENT (7.3 mmol/kg dry weight; 0.003), and highest in the CG and MTG—with both having significantly higher concentrations than the GP (5.5 mmol/kg dry weight; p = 0.0013 and 0.0019, respectively) as well as the SN.

### Mn

In controls, Mn levels were significantly higher in the PUT (45.7 µmol/kg dry weight) in comparison to several other investigated regions, including the MCX (17.8 µmol/kg dry weight; p < 0.0001; see Supplementary Figure 2), SCX (20.0 µmol/kg dry weight; p < 0.0001), CG (22.5 µmol/kg dry weight; p = 0.046), MFG (19.5 µmol/kg dry weight; p = 0.0005), MTG (19.6 µmol/kg dry weight; p = 0.0005), and ENT (20.9 µmol/kg dry weight; p = 0.005). Mn concentrations were also higher in the GP (33.0 µmol/kg dry weight) compared to the MCX (p = 0.0006), SCX (p = 0.003), MFG (p = 0.04), and MTG (p = 0.04) and in the CB (37.9 µmol/kg dry weight) compared to the MFG (p = 0.003), MTG (p = 0.003), and ENT (p = 0.03).

As in the controls, Mn levels were highest in the PUT (40.6 µmol/kg dry weight) in HD cases, being significantly higher compared to the MCX (21.3 µmol/kg dry weight; p = 0. 011; see Supplementary Figure 3), SCX (19.9 µmol/kg dry weight; p = 0.0002), SN (19.8 µmol/kg dry weight; p = 0.0007), MFG (21.6 µmol/kg dry weight; p = 0.02), MTG (21.0 µmol/kg dry weight; p = 0.007), and ENT (20.8 µmol/kg dry weight; p = 0.005). Levels in the CB (36.5 µmol/kg dry weight) were also again higher in comparison to those in the MFG (p = 0.03), MTG (p = 0.0104), ENT (p = 0.008), as well as those in the MCX (p = 0.02), SCX (p = 0.0003), and SN (p = 0.0011). Levels were fairly consistent among the other regions in HD cases.

### Fe

In controls, Fe levels were highest in the PUT (14.2 mmol/kg dry weight), being significantly higher in comparison to the CG (4.3 mmol/kg dry weight; p = 0.0006; see Supplementary Figure 2), MFG (4.8 mmol/kg dry weight; p = 0.02), HP (3.8 mmol/kg dry weight; p < 0.0001), and ENT (4.4 mmol/kg dry weight; p = 0.0006). Fe levels were also higher in the GP compared to the CG (p = 0.005), HP (p < 0.0001), and ENT (p= 0.005), and in the SN (4.3 mmol/kg dry weight) compared to the HP (p = 0.013). Concentrations in the other regions were quite consistent.

A similar pattern was present in the HD cases; Fe PUT concentrations (16.8 mmol/kg dry weight) were significantly higher in comparison to the CG (4.5 mmol/kg dry weight; p = 0.002; see Supplementary Figure 3), HP (3.6 mmol/kg dry weight; p < 0.0001), and ENT (4.4 mmol/kg dry weight; p = 0.0006). GP Fe levels were also significantly higher than those in the HP (p = 0.003). Other regions showed fairly consistent Fe concentrations.

### Cu

In controls, Cu concentrations were fairly consistent; however, there were significantly increased levels in the CB in comparison to the MCX (319.8 µmol/kg dry weight; p = 0.005; see Supplementary Figure 2), HP (286.0 µmol/kg dry weight; p = 0.0104), and ENT (327.8 µmol/kg dry weight; p = 0.013) as well as higher levels in the SN (579.5 µmol/kg dry weight) compared to the MCX (p = 0.004).

Cu levels were also quite consistent across HD case regions, although they were lower in the HP (256.1 µmol/kg dry weight) compared to the PUT (455.0 µmol/kg dry weight; 0.002; see Supplementary Figure 3), SN (361.8 µmol/kg dry weight; p 0.03), and CB (p = 0.0001).

### Zn

In controls, Zn levels were highest in the HP (1310.1 µmol/kg dry weight), being significantly higher in comparison to levels in the MCX (808.4 µmol/kg dry weight; p = 0.0005; see Supplementary Figure 2), SCX (913.8 µmol/kg dry weight; p = 0.008), GP (p < 0.0001), and SN (846.7 µmol/kg dry weight; p = 0.047). Zn levels in the control CG (1052.3 µmol/kg dry weight) were also significantly higher than in the MCX (p = 0.03) and GP (p = 0.002).

In cases, Zn levels were highest in the PUT, being significantly higher than in the MCX, (897.3 µmol/kg dry weight; p = 0.03; see Supplementary Figure 3), SCX (967.4 µmol/kg dry weight; p = 0.007), GP (p = 0.0102), and SN (761.0 µmol/kg dry weight; p = 0.0014). They were lowest in the SN, being significantly lower compared to the CG (1247.5 µmol/kg dry weight; p = 0.0008), MTG (1304.0 µmol/kg dry weight; p = 0.0003), HP (1208.6 µmol/kg dry weight; p = 0.0105), and ENT (9.7 µmol/kg dry weight; p = 0.02) as well as the PUT. Levels in the MCX, SCX, and GP were very similar, whilst the MFG, MTG, CB (1118.6 µmol/kg dry weight), HP and ENT showed fairly consistent Zn concentrations to one another.

### Se

In controls, Se levels were lowest in the SCX (mean 12.5 µmol/kg dry weight), being significantly lower than in the MTG (mean 17.0 µmol/kg dry weight; p = 0.03; see Supplementary Figure 2) and CB (28.2 µmol/kg dry weight; p = 0.04). Otherwise, control Se concentrations were fairly consistent across regions.

In cases, MTG (11.8 µmol/kg dry weight) and CB (12.4 µmol/kg) Se concentrations were significantly higher than in the MCX (11.1 µmol/kg dry weight; p = 0.0452 and p = 0.006, respectively; see Supplementary Figure 3), GP (8.8 µmol/kg; p = 0.0096 and p = 0.0011, respectively), and SN (8.7 µmol/kg; p = 0.007 and p = 0.0007, respectively), but were otherwise consistent.

## Supplementary Figure 1: Metal Concentrations Across Control Brain Regions

## Supplementary Figure 2: Metal Concentrations Across HD Case Brain Regions

## Supplementary Discussion—Application of Bradford-Hill Criteria to Se Findings

The Bradford Hill Criteria were applied to the case–control data on Se levels; these criteria are defined by nine points: *strength* (effect size), *consistency* (reproducibility), *specificity*, *temporality*, *biological* *gradient* (dose–response relationship), *plausibility*, *coherence*, *experiment*, and *analogy*. Here, the *strength* of our Se findings is indicated by the presence of p-values < 0.05, being <0.01 in most regions and as low as <0.001 in the PUT; risk ratios ≥2.0, with most being ≥3.0 and many reaching ≥8.0, indicating strong associations. The *consistency* of our Se findings is supported by a previous report of Se decreases in selected regions of the brain investigated in the current study (the CB, CG, and PUT), as well as indications that Se supplementation can attenuate motor deficits and neuronal loss in HD mouse models (1, 2). *Specificity* is demonstrated by the differences observed between Se findings and alterations in essential metals investigated in the current study; no other analyte showed significant changes across every brain region, with most only showing regionally localised perturbations. The *temporality* of our Se findings cannot be inferred from the current data, but further studies investigating Se concentrations in preclinical HD and various clinical stages of HD (based on Vonsattel grades) could inform on this aspect of the data. The presence of a *biological gradient* is supported by HD animal model studies in which neuronal and clinical signs of the disease appear to be attenuated by supplementation of Se and resultant attenuation of diminished Se levels (1, 2)—although this requires further investigation in humans. The *plausibility* of a diminished Se–HD pathology relationship is supported by such supplementation studies, as well reports of impaired motor function and cognitive impairment resulting from Se deficiency (3, 4); the role of Se as an essential cofactor for many important antioxidant enzymes and selenoproteins suggests potential pathogenic mechanisms via increased mitochondrial dysfunction and oxidative stress, deficient neuronal signalling, and disruption of energetic pathways in the brain. The *coherence* of the proposed Se–HD association is supported by previous reports of Se deficiency in the HD brain, as well as in some other neurodegenerative diseases, and reports of oxidative stress and mitochondrial dysfunction in HD, which may be related to such deficiencies (5, 6); our reported data do not conflict with previous reports in HD. Fulfilment of the *experiment* criteria are supported by the supplementation studies performed in HD mouse models, but would be more strongly supported by human intervention studies. Fulfilment of the *analogy* criteria is given by reports of dietary Se deficiency leading to cognitive impairment and motor dysfunction—both of which occur in HD. Taken together, the fulfilment of the majority of the Bradford–Hill criteria, as well as the strong risk ratios and effect sizes seen for the Se data, indicate a good level of epidemiological evidence in support of a potential causal relationship between diminished cerebral Se levels and HD.

**References**

1. Lu Z, Marks E, Chen J, Moline J, Barrows L, Raisbeck M, et al. Altered selenium status in Huntington's disease: neuroprotection by selenite in the N171-82Q mouse model. Neurobiol Dis. 2014;71:34-42.

2. Abdelfattah MS, Badr SEA, Lotfy SA, Attia GH, Aref AM, Abdel Moneim AE, et al. Rutin and Selenium Co-administration Reverse 3-Nitropropionic Acid-Induced Neurochemical and Molecular Impairments in a Mouse Model of Huntington's Disease. Neurotox Res. 2020;37(1):77-92.

3. Shreenath AP, Ameer MA, Dooley J. Selenium Deficiency. StatPearls. Treasure Island (FL)2022.

4. Pillai R, Uyehara-Lock JH, Bellinger FP. Selenium and selenoprotein function in brain disorders. IUBMB Life. 2014;66(4):229-39.

5. Kumar A, Ratan RR. Oxidative Stress and Huntington's Disease: The Good, The Bad, and The Ugly. J Huntingtons Dis. 2016;5(3):217-37.

6. Bjorklund G, Shanaida M, Lysiuk R, Antonyak H, Klishch I, Shanaida V, et al. Selenium: An Antioxidant with a Critical Role in Anti-Aging. Molecules. 2022;27(19).

## Supplementary Figure 3: Individual Sample Profiles

Graphs show mean deviation of each case and control, normalised to the control mean. Error bars show mean ± SD.
